# Forecasting COVID-19 new cases using NBEATS deep learning and mobility data

**DOI:** 10.1371/journal.pone.0350264

**Published:** 2026-06-29

**Authors:** Amril Nazir, Mohammad Shorfuzzaman, Muhammad Lujaini Lotfi, Firuz Kamalov, Sufian Badawi, Maen Takruri, Abdul-Halim Jallad

**Affiliations:** 1 Department of Information Systems and Technology Management, Zayed University, Abu Dhabi, United Arab Emirates; 2 Department of Software Engineering, College of Engineering and Advanced Computing, Alfaisal University, Riyadh, Saudi Arabia; 3 AnalytiCray Ltd, Kuala Lumpur, Malaysia; 4 Canadian University Dubai, Dubai, United Arab Emirates; 5 Department of Autonomous Systems, Faculty of Artificial Intelligence, Al-Balqa Applied University, Al-Salt, Jordan; 6 College of Engineering and Technology, American University of the Middle East, Egaila, Kuwait; 7 Department of Electrical Engineering, United Arab Emirates University, Al Ain, United Arab Emirates; Newcastle University, UNITED KINGDOM OF GREAT BRITAIN AND NORTHERN IRELAND

## Abstract

COVID-19 is a highly contagious disease transmitted primarily through human contact. Therefore, understanding population mobility is essential for predicting COVID-19 case trends. In this paper, we propose a novel deep learning approach for forecasting new COVID-19 cases using a neural architecture called Neural Basis Expansion Analysis for Interpretable Time Series (N-BEATS). The N-BEATS model effectively handles long input sequences and large output horizons without information loss or increased computational complexity. We compare the performance of N-BEATS with a state-of-the-art benchmark model, LSTM-Markov, across four major countries: the United States, the United Kingdom, Russia, and Brazil. Three distinct COVID-19 datasets from Google, Apple, and Our World in Data (OWID) were used in this study. Incorporating Google and Apple mobility data as covariates enhances both the accuracy and interpretability of the N-BEATS model. Our results show that N-BEATS consistently outperforms LSTM-Markov across all datasets and countries, consistently yielding lower Root Mean Squared Error (RMSE) and Mean Absolute Percentage Error (MAPE). Furthermore, the N-BEATS model with covariates outperforms its counterpart without covariates, indicating that mobility data provide substantial value for forecasting new COVID-19 cases. Overall, this study demonstrates the effectiveness of the N-BEATS architecture in capturing pandemic dynamics and offers valuable insights for policymakers and public health officials in managing future outbreaks.

## 1 Introduction

On March 11, 2020, the World Health Organization (WHO) declared COVID-19 a global pandemic [1], as the virus rapidly spread across the world. Most countries subsequently experienced two major waves of the pandemic. The initial wave occurred around April and May 2020, followed by a significantly more severe second wave from September to December 2020. The severity of the second wave was marked by a dramatic rise in the number of infections and deaths. For instance, on April 1, 2020, the U.S. government reported that infections and deaths increased from 255,000 and 5,000 respectively, to 20.3 million infections and 380,000 deaths at the end of December 2020 [2]. A similar trend was observed in other nations; for example, the U.K. reported a surge from 33,568 cases and 5,127 deaths in early April 2020 to 2.6 million and 93,317 at the end of December 2020, respectively. Since then, distinct COVID-19 variants (denoted as α, β, γ, and δ) have emerged in different countries, triggering successive waves that appeared locally before spreading geographically. By November 2022, the number of confirmed cases had risen to approximately 640 million, with deaths surpassing 6.6 million worldwide [2]. Many vaccination programs were launched in response to COVID-19; however, these vaccines were not fully protective against certain variants. The rapid mutation of the virus kept the pandemic a global concern, despite the implementation of robust vaccination campaigns [3]. In response, many countries were compelled to enforce strict measures to control the pandemic and transition toward epidemic management [4]. Human mobility—a key factor in social interaction and interpersonal contact [5]—was a critical determinant of COVID-19 transmission. Consequently, travel restrictions were widely implemented to limit human-to-human spread [5]. These mobility restrictions effectively curbed transmission rates and were associated with a decrease in infection cases [6].

Although multiple vaccines have been developed and widely administered, significant concerns about continued transmission and the emergence of new variants persist. Therefore, developing accurate strategies to mitigate the spread of COVID-19 and to establish effective contingency policies remains crucial. In this context, forecasting future epidemic trends plays a vital role in helping authorities design timely interventions, allocate medical resources efficiently, and minimize the pandemic’s societal impact. Nevertheless, it should be noted that the OWID dataset used in this study contains unspecified or missing vaccination data for most countries and time periods, which may introduce bias and limit the model’s ability to fully capture the temporal effects of vaccination on pandemic dynamics [7]. For this reason, vaccination data were excluded. Second, the mobility data obtained from Google [8] and Apple [9] may not fully represent entire populations, as they reflect only the behaviors of individuals with smartphones and internet connectivity, potentially leading to demographic and regional biases. Third, variations in data quality across countries—such as differences in testing capacity, case definitions, and reporting transparency—may affect the comparability of results. Finally, changes in pandemic reporting strategies over time, including retrospective adjustments and delayed updates, could introduce additional uncertainty into the forecasts.

In this study, we propose a novel approach for forecasting the spread of COVID-19. The proposed method integrates Neural Basis Expansion Analysis for Interpretable Time Series (N-BEATS) with mobility data from Apple’s COVID-19 Mobility Trends Reports and Google’s COVID-19 Community Mobility Reports to achieve state-of-the-art forecasting performance. The data used in this study were obtained from three publicly available COVID-19 datasets: Google [8], Apple [9], and Our World in Data (OWID). We focused on four major countries—the United States, the United Kingdom, Brazil and Russia—that were among the most severely affected by the pandemic. The performance comparison between the proposed method and two benchmark models, namely LSTM and LSTM-Markov, demonstrates the superior effectiveness of our approach. Although N-BEATS is a relatively simple deep learning architecture, it is highly expressive and requires no specific feature engineering or input scaling. This property enables the exploration of the intrinsic potential of a pure deep learning framework for time series forecasting. Consequently, our approach achieves better performance than previous state-of-the-art models.

The main contributions of this paper are the following:

**Superior forecasting performance:** We applied the N-BEATS neural architecture, which can handle long input sequences and large output horizons, to forecast daily COVID-19 new cases. We showed that N-BEATS achieved improved accuracy over the existing benchmark model LSTM-Markov, which suffers from the vanishing gradient problem and high computational complexity.
**Integration of comprehensive mobility data:** We incorporated mobility data from Google and Apple as covariates into the N-BEATS model, which enhanced its accuracy. We demonstrated that mobility data can provide valuable insights into the factors influencing the spread of COVID-19 and help policymakers make more informed decisions.
**Simple yet highly effective method:** We proposed a simple and easy-to-implement method that can forecast COVID-19 new cases with high accuracy. Our method does not require any complex preprocessing or feature engineering, and can be applied to any country or region with available data.

The rest of the paper is arranged into several sections as follows: Section 2 covers the previous related research work, Section 3 elaborates on the proposed deep learning method and the creation of the materials used to achieve the research objective, Section 4 covers the results of applying the deep learning approach to the four countries’ data and its discussion of the findings of the results, and finally section 5 cover the conclusions and the proposed future research.

## 2 Related work

COVID-19 is a serious pandemic. No one knows what the pandemic’s future holds. In this difficult period, describing COVID-19 impact is vital. Surveys and reviews about COVID-19 are needed to be conducted from research angles, and thoroughly reviewed on how the COVID-19 affects in all dimensions of mobility such as transportation, lifestyle, tourism, air travel, and environment. The study will examine the effect of COVID-19’s impacts on transportation mobility and lifestyle to discover changes, negative effects, and new opportunities. During pandemic, limitations on mobility have changed people lifestyles. When people are stringently quarantined at home, the tendency to acquire stress and unhappiness is higher than usual due to less physical and social interaction among friends and family [10].

Recently, André de Palma [11] has conducted a study on the effects of Covid-19, 18 months since the outbreak. The outbreak has caused vast of population lost their lives. By September 2021, it was estimated almost 4.5 million deaths worldwide. The author has stressed that besides the public health crisis, the pandemic has affected globally to the enormous economy consequences and development progression significantly. The severity of the disruption could lead potentially to a long-term impact for many countries to recover. As uncertainties have loomed over, drastic changes in people’s mobility and lifestyle could also contribute to negative behavioral changes. The COVID-19 has changed the lifestyle of many people, due to the limited mobility during stringent quarantine at home. As the consequence, several negative outcomes have been identified that bring about to cardiovascular disease, obesity, and other health problems [12].

On the mobility of traffic perspective, drastic reduction on traffic accident can be seen due to the limited personal mobility has been imposed on the road. Restrictions on traffic has implied a sharp reduction in road traffic. Since the outbreak, about 74% fell from the total number of traffic accidents, which subsequently reduces drastically the number of fatalities. The reduction of traffics on road also fostered a positive environmental improvement as well. Traffic restrictions have improved air quality due to the reduction of air pollutants being discharged into the atmosphere. It also reduces other environmental problems such as noise and greenhouse gas emissions significantly, which implies positive impact on human health [13]. In aviation industry also has been heavily weighted by the stringent air travel restrictions. Since the declaration of COVID-19 as pandemic by the World Health Organization (WHO) [1], many airlines debilitated by the closure of the borders and airports. Kaitano Dube et al. [14] has pointed the number of commercial flights in April 2022 has plummeted to the lowest daily flights in many countries. It was estimated that the daily flight averaged merely 24,049 flights. It resulted in several airlines and airports to ratings downgrades, liquidation, and bankruptcy.

Researchers have been inspired to investigate new approaches for predicting demand for travelers in unpredictable circumstances due to the influence of COVID-19 on the tourism sector. Wu et al. [15] introduced a transparent architecture that integrates several types of multivariate time series data, including historical tourist volume, COVID-19 data, search engine data, and weather data, using a deep learning model called temporal fusion transformers (TFT). The researchers used a differential evolution technique to tune the hyperparameters of TFT and proposed a novel approach for analyzing search engine data using variational mode decomposition. Their methodology has the capability to provide precise and comprehensible predictions of tourist volume for three picturesque locations in China. Wu et al. [16] also developed a hybrid model that combines a convolutional neural network (CNN) and a long short-term memory (LSTM) network to predict the daily power use of hotels in China during the COVID-19 epidemic. The researchers used a genetic algorithm to carefully choose the most effective input characteristics and parameters for their model. They provided evidence that their model surpasses many benchmark models in terms of both accuracy and resilience. Wu et al. [15] conducted a study where they used a deep neural network (DNN) model to forecast the monthly influx of foreign tourists in China. The researchers used a particle swarm optimization approach to fine-tune the DNN model and conducted a comparative analysis with other conventional models, including autoregressive integrated moving average (ARIMA) and support vector regression (SVR). The researchers discovered that the Deep Neural Network (DNN) model is capable of capturing the intricate and non-linear patterns of tourist demand. Furthermore, the DNN model outperforms standard methods in terms of performance.

Although several studies have compared deep learning methods with conventional statistical approaches such as ARIMA, the incorporation of the ARIMA model as an additional baseline for comparison with existing traditional models, namely the LSTM and LSTM-Markov models, is not sufficiently compelling for this study. By design, ARIMA employs an auto-regressive framework structured around linear combination algorithms, which limits its capacity to capture the non-linear and highly volatile characteristics of COVID-19 new case data [17]. Given the dynamic nature of pandemic time series, this linearity makes ARIMA unsuitable for modeling abrupt fluctuations, structural changes, and non-stationary behavior. Consequently, many researchers have favored more sophisticated architectures—such as the LSTM used in LSTM-Markov analysis or deep learning approaches like N-BEATS—that can better represent complex temporal dependencies and nonlinear dynamics. As highlighted by Ma [18], the ARIMA model performs poorly in long-term COVID-19 forecasting due to its inability to adapt to evolving epidemic patterns. Therefore, this study maintains the LSTM-Markov model as the primary baseline for comparison, providing a more appropriate and theoretically justified foundation for evaluating the N-BEATS architecture.

Inactivity and overuse of mobile phones and computers cause heart disease, obesity, and other health concerns. [12] Economic and social mobility could be affected. COVID-19 alters departure times, modes, and routes. In the long term, carless households will buy cars. Governments must prepare. Socioeconomic activity decline and transportation needs (air travel, inter-urban, and urban), While Negative yet positive: (8%) worldwide coal demand in comparison to 2019, (5%) oil demand, (2%) gas consumption, (20%) power demand, (74.3%) road accidents. [13] Total V.M.T. and work-related travels have reduced since COVID-19, but total trips have grown. Cold beginnings may increase. Without help, This might result in more short drives and chilly starts on local roads using engines with internal combustion. without the appropriate infrastructure to support renewable energy transition.

The effect of the Coronavirus lockdown on people’s travel behavior in Sharjah, U.A.E. (retail and leisure, supermarket and transit stations, pharmacy, parks, workplace, and residential) is studied in [19], using statistical analysis to identify the probable cause-and-effect correlations. The correlation between travel behavior with air quality and energy utilization. Parks businesses were impacted most, while grocery and pharmacy were the least. By March 2021, when most legal limitations were abolished, all sectors’ mobility was below the pre-pandemic benchmark except retail, leisure, groceries, and pharmacy. The lockout reduced NO2 levels compared to 2019. Location and surroundings affected ground-based air quality measurements. At a Sharjah crossroads, less traffic improved air quality. Throughout the outbreak, wind direction and speed did not affect air quality. All mobility types except residential had a positive association between transportation and NO2 trends.

The objective of the study conducted by Luo et al. (2023) [20] is to investigate the viability of using a time-series clustering methodology to examine the patterns of the COVID-19 pandemic. The authors use dynamic temporal warping distance and hierarchical clustering techniques to categorize time series data of daily new cases and fatalities from various nations into four distinct patterns. Additionally, they investigate the potential determinants that impact the development of cluster patterns, including the geographical positioning and demographic composition of the population. The literature assessment of the article exhibits both advantages and disadvantages. The literature review offers a comprehensive and succinct summary of the current research that uses time-series clustering for COVID-19 data processing, highlighting its favorable aspects. Furthermore, it discerns the gaps in research and the impetus behind the suggested approach. However, the literature study does not address the constraints and difficulties associated with the time-series clustering technique. Furthermore, a comprehensive analysis of the suggested approach in relation to other established methods, namely in terms of performance, benefits, and drawbacks, is not provided.

Six distinct deep learning classifiers are applied to the COVID-19 dataset for India infections by [21] and aimed to identify the best one that provides vital information for combatting the pandemic and may even help public health. They used RMSE to evaluate the effectiveness of every time series to model. Despite that, the two methods, ARIMA and TBAT, are suggested as they generate the best overall results; ARIMA is incapable of forecasting nonlinear problems.

LSTM [22] is a Recurrent Neural Network (R.N.N.) is a subclass of neural network (N.N.) that conducts temporal correlations and avoids memory difficulties. It consists of memory cells, which retain information for an extended period. Several components known as gates either discard, store, or modify the information in the cells. Three gates comprise LSTM: input gate, output gate, and forget gate. The forget gate determines whether data should be deleted or stored in the cell. This operation applied the hyperbolic tangent or sigmoid functions. The sigmoid layer uses the current input and the previous hidden state to provide a 0–1 output.

LSTM-Markov [23] proposes an LSTM model linked with the learning algorithm since current LSTM models cannot properly foresee data deviation difficulties. The model was trained using U.S., Brazil, UK, and Russia case data. Using a Markov model-correcting LSTM model, predict each country’s reported cases by 20 February 2021. We will evaluate our model using R2, RMSE, and error rate. According to the estimate, the number of cases will stabilize, and Britain’s pandemic to be under control by February 2021. Still, it will continue to rise in the U.S., Brazil, and Russia. The suggested model’s predicting curve is comparable to the real pandemic graph. The R2 values are all around 1, the average RMSE is only 40% of the LSTM model, and the mean error is decreased by more than 75%. LSTM-Markov is more accurate than LSTM. Comparing the LSTM-Markov error rate against the LSTM error rates, the LSTMS model shows higher results. The LSTM-Markov model improves more than applying LSTM alone. The LSTM-Markov model can reliably predict verified circumstances. Although predicted results may improve government decision-making in drafting appropriate measures and applicability, the results “Root Mean Square Error" (RMSE) [24] seem high in the proposed model results.

Jeng et al. (2021) [25] provide a new approach in their work, which integrates wastewater-based surveillance (WBS) with a copula time-series model to predict COVID-19 outcomes. The authors use World Bank Statistics (WBS) data from 12 cities in China to calculate the infection rate and the reproduction number of COVID-19. In addition, a copula time-series model is used to accurately represent the interdependence and individual distribution of the WBS data. The authors conduct a comparative analysis of their technique against other models, including ARIMA, LSTM, and GARCH. The results demonstrate that their method outperforms the others in terms of accuracy and error reduction. The literature review of the study contains some merits and demerits. The literature review offers a concise and clear summary of the current research using WBS and time-series models for COVID-19 analysis. Additionally, it clearly states the gap in research and the motivation behind the suggested approach. Be that as it may, the work does not address the constraints and difficulties associated with the WBS and copula time-series methods. Furthermore, a comparative analysis of the suggested approach with other established methodologies in terms of performance, benefits, and drawbacks, would have been welcomed.

The work done by Kamalov et al. (2022) [26] provides a comprehensive examination and evaluation of the existing studies that use deep learning techniques for the purpose of predicting the spread and impact of Covid-19. The authors categorize the research into four groups according to the nature of the data and the objective of the prediction: verified cases, fatalities, hospitalizations, and movement. In addition, they evaluate the performance, benefits, and constraints of several deep learning models, including recurrent neural networks, convolutional neural networks, and graph neural networks. The research delineates the primary obstacles and prospective pathways for Covid-19 prediction via deep learning, including issues of data integrity, ability to interpret, uncertainty, and generalization. The study offers a thorough and organized examination of the most advanced deep learning methods for predicting Covid-19 and their potential use in making decisions related to public health. The study presents a comprehensive literature review that mentions both advantages and disadvantages. Subsequently, the literature review offers a thorough and methodical summary of the present research that employs deep learning techniques for Covid-19 prediction. Additionally, it categorizes the research into four distinct groups, taking into account the nature of the data and the specific forecasting objective. This classification facilitates the comparison and analysis of the effectiveness, benefits, and constraints of various deep learning models. However, the literature review falls short in conducting a thorough assessment of the quality and validity of the sources and does not specify what exactly is lacking in the literature. Furthermore, the research topic and the objective of the literature review are not explicitly articulated, which impedes the evaluation of its pertinence and impact in the area.

The study authored by Guo et al. (2021) [27] introduces a novel method named Weibo COVID-19 Trends (WCT), which aims to enhance the precision and practicality of Google Flu Trends (GFT) in predicting the number of COVID-19 cases in China. The authors use a genetic algorithm to determine the most advantageous combination of keywords for the purpose of filtering COVID-19-related postings from Sina Weibo, a widely used social media site in China. The performance of WCT, GFT, and the officially recorded cases of COVID-19 in Wuhan, the epicenter of the epidemic, were compared. They discover that WCT can surpass GFT and the official reports in terms of correlation, inaccuracy, and peak estimate. Additionally, they demonstrate that the combination of a wavelet coherence transform (WCT) and an ARIMA model may enhance the accuracy of COVID-19 case predictions for a period of one to four weeks. The study showcases the use of data from social media to augment the monitoring and forecasting of contagious illnesses.

To name some other machine learning methods that are used to predict the COVID-19 spread on mobility, we found a few like the Holt-Winters additive model (HWAAS) [28], Auto time series prediction (Facebook’s “Prophet”) [29], the quadrant method (Trigonometric seasonal formulation, Box–Cox transformation, ARMA errors, and Trend component) TBAT [30], Auto-Regression-Integrated-Moving-Average ARIMA [31], the trend and the seasonality stacks based N-BEATS [32], and Probabilistic Auto-Recurrent Network Forecasting (DeepAR) [33], deep learning based timeseries forcasting [34]. Other authors have created methods to predict the incidence of COVID-19 cases based on data on population mobility. Some base their research on the “COVID-19 Community Mobility Reports” published by Google [35–39]. Nonetheless, very little work has been done on using mobility data or considering people’s mobility when predicting new daily cases.

In this Study, LSTM, LSTM-Markov, and N-BEATS deep learning model are applied with two sets of parameters and applied in 3 COVID-19 datasets and compared with the results of LSTM and LSTM-Markov results trying to reach a more precise model with fewer RMSE rates.

## 3 Methods

### 3.1 N-BEATS model

Sophisticated time-series forecasting problems often require the use of complex neural network architectures. In this study, we employ the N-BEATS model—implemented through the DARTS API—to forecast COVID-19 cases. N-BEATS, short for **Neural Basis Expansion Analysis for Time-Series Forecasting** [40], is a state-of-the-art deep learning model designed to be simple, generic, and highly expressive. Its simplicity lies in the fact that it requires no feature engineering or input scaling, allowing researchers to focus on the pure representational power of its deep learning framework.

**Fig 1:**
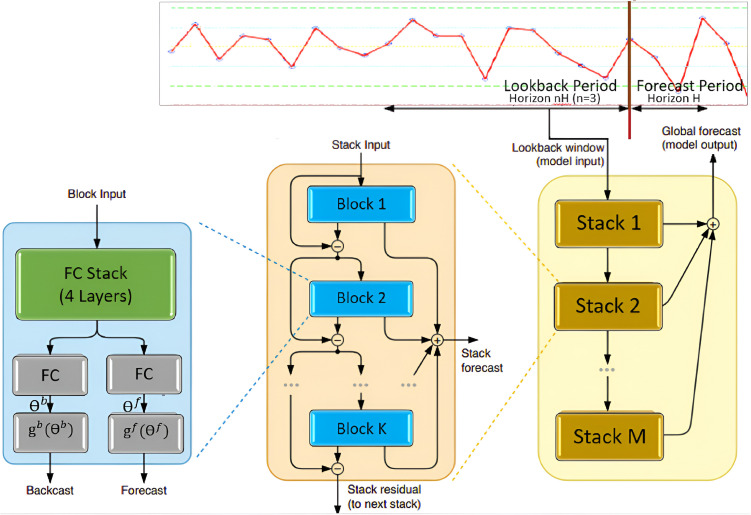
The architecture of the N-BEATS model.

The N-BEATS model is formulated through a composition of deep learning algorithms tailored to handle non-linear and non-stationary time-series data. This design enables the model to capture intricate temporal dependencies and seasonal variations without relying on domain-specific assumptions. Fig 1 illustrates the model structure, which consists of multiple blocks arranged sequentially in stacks. Each block, indexed by (λ), receives an input $\left(x_{\lambda }\right)$ and produces two output vectors: the backcast $\left({\hat{x}}_{\lambda }\right)$ and the forecast $\left({\hat{y}}_{\lambda }\right)$. In the first block, $\left(x_{\lambda }\right)$ represents the overall input data, whereas in subsequent blocks, $\left(x_{\lambda }\right)$ corresponds to the residual output from the previous block. The **lookback period** defines the input window size over which the model learns temporal patterns, while the **forecast period** represents the time horizon for future prediction.

Each N-BEATS block adopts a **forked architecture** to ensure both interpretability and extensibility. It consists of multiple fully connected layers with ReLU activations, used to estimate basis expansion coefficients for both forecast and backcast components. The blocks are organized into stacks following the **doubly residual stacking** principle, where forward and backward residual links operate across deep layers of fully connected networks. This principle allows the model to iteratively refine its predictions by passing residual information through both forecast and backcast pathways, giving rise to the term “doubly residual stacking.”

Each stack may contain multiple layers that share the coefficients $\left(g^{b}\right)$ and $\left(g^{f}\right)$, representing the backcast and forecast coefficients, respectively. Forecasts are generated and accumulated hierarchically, producing interpretable outputs across the deep neural structure. Every block produces two outputs—$\left(\hat{y}\right)$ and $\left(\hat{x}\right)$—which correspond to the **forward forecast** and **backward backcast** components. The internal structure of each block is composed of two primary parts:

- **Fully Connected (FC) Network:** This component transforms the input vector $x_{\lambda }$ through a sequence of fully connected layers, each followed by a ReLU activation, to extract nonlinear representations of temporal dependencies. The operation is defined as:
  $$\begin{array}{cccc} h_{\lambda ,1} & =FC_{\lambda ,1}(x_{\lambda }), & \; & h_{\lambda ,2}=FC_{\lambda ,2}(h_{\lambda ,1})\\ h_{\lambda ,3} & =FC_{\lambda ,3}(h_{\lambda ,2}), & \; & h_{\lambda ,4}=FC_{\lambda ,4}(h_{\lambda ,3})\\ \theta_{\lambda }^{b} & =\text{LINEAR}(h_{\lambda ,4}), & \; & \theta_{\lambda }^{f}=\text{LINEAR}(h_{\lambda ,4}) \end{array}$$ (1)
  Here, $h_{\lambda ,1\ldots 4}$ represent the successive hidden activations, while $\theta_{\lambda }^{b}$ and $\theta_{\lambda }^{f}$ are the backcast and forecast expansion coefficients, respectively. Each fully connected layer applies a ReLU activation:
  $$\begin{array}{c} h_{\lambda ,i}=ReLU(W_{\lambda ,i}h_{\lambda ,i-1}+b_{\lambda ,i}) \end{array}$$ (2)
  The linear layer performs the projection operation:
  $$\begin{array}{c} \theta_{\lambda }^{f}=W_{\lambda }^{f}h_{\lambda ,4}+b_{\lambda }^{f} \end{array}$$ (3)
- **Basis Projection Layer:** In this part, the learned coefficients $\theta_{\lambda }^{b}$ and $\theta_{\lambda }^{f}$ are projected onto sets of **basis functions** $g^{b}$ and $g^{f}$, respectively:
  $$\begin{array}{c} {\hat{x}}_{\lambda }=g^{b}(\theta_{\lambda }^{b}),\;{\hat{y}}_{\lambda }=g^{f}(\theta_{\lambda }^{f}) \end{array}$$ (4)
  These projections generate the **backcast** (reconstructed past signal) and **forecast** (predicted future signal), which work together to ensure residual information is minimized and forecast accuracy is maximized.
  The stacking and residual operations across blocks are expressed as:
  $$\begin{array}{c} \hat{y}=\sum_{\lambda }{\hat{y}}_{\lambda },\;x_{\lambda }=x_{\lambda -1}-{\hat{x}}_{\lambda -1} \end{array}$$ (5)
  The forward and backward coefficients produce their respective outputs through basis functions as follows:
  $$\begin{array}{c} {\hat{y}}_{\lambda }=\sum_{i=1}^{\dim(\theta_{\lambda }^{f})}\theta_{\lambda ,i}^{f}V_{i}^{f}\;{\hat{x}}_{\lambda }=\sum_{i=1}^{\dim(\theta_{\lambda }^{b})}\theta_{\lambda ,i}^{b}V_{i}^{b} \end{array}$$ (6)
  where $V_{i}^{f}$ and $V_{i}^{b}$ denote the forecast and backcast basis vectors, respectively.

Following a similar strategy to [23], hyperparameters were optimized through a targeted trial-and-error approach rather than exhaustive grid search or Bayesian optimization, which would have been computationally prohibitive across multiple countries and datasets. Variations in input chunk length were systematically evaluated, as this parameter had the greatest influence on capturing country-specific temporal dynamics. The best values were selected based on RMSE and MAPE performance, while the optimal time-step was determined by jointly minimizing RMSE, MAPE, and training loss. For the remaining parameters—such as number of stacks (30), number of blocks per stack (1), number of layers (4), layer widths (256), expansion coefficient dimension (5), polynomial degree of trend (2), and dropout (0.0)— we adopted the default configuration provided by the Darts N-BEATS implementation. This ensured reproducibility, alignment with prior literature, and reduced risk of overfitting. The final parameter specifications are summarized in Table 7. Hyperparameter selection was carried out using a targeted trial-and-error procedure rather than exhaustive grid search or Bayesian optimization, which would have been computationally prohibitive across multiple countries and datasets. Particular attention was given to input chunk length and forecast time-step settings, as these parameters had the strongest influence on the model’s ability to capture temporal dynamics. Candidate settings were assessed using RMSE, MAPE, and training loss, and the final configuration was selected based on overall predictive performance. The *num of layers* parameter is an important variable that specifies the number of fully connected layers preceding the final forking layers in each block of every stack. The *input chunk length* parameter defines the number of time steps used as input for the current model, representing a predetermined range of upcoming days. The *dropout* parameter indicates the probability of randomly deactivating neurons in the fully connected layers to prevent overfitting. The *epochs* parameter determines the total number of iterations for model training. The *batch size* parameter specifies the number of samples processed before updating the model’s weights. The *learning rate* parameter controls the magnitude of weight updates during optimization. The *num of stacks* parameter defines the number of stacks that together form the overall architecture of the model, each composed of multiple blocks designed to capture various patterns in the data.

## 4 Results and discussion

The emergence of COVID-19 was highly unprecedented, leaving only limited historical references for modeling. Since the N-BEATS forecasts rely heavily on patterns within the lookback window, this constraint often led to deviations that manifested as outliers [41]. Furthermore, irregularities in the reported data—caused by delays in reporting, sudden shifts in government policies, or testing surges—produced abrupt jumps or dips, which subsequently propagated into the model’s forecasts. In addition to data-related factors, the intrinsic characteristics of the N-BEATS architecture also contribute to forecast volatility. The model’s purely data-driven design treats sharp fluctuations as signal rather than noise, and its doubly-residual stacking may magnify small deviations, particularly at shorter forecast horizons. While the inclusion of mobility covariates improved average performance, it also introduced additional variance in periods where mobility patterns were weakly correlated with transmission dynamics (e.g., holidays or sudden lockdowns). Importantly, many of the detected outliers coincided with the onset of major pandemic waves or rapid intervention changes, suggesting that these deviations are not purely artifacts of model error but rather reflections of real-world regime shifts.

### 4.1 Data source

The statistics used in this study are collected from 3 different sources, including the data from Our World in Data [42] by the University of Oxford, Apple’s Covid-19 Mobility Trends Reports [9], and Google’s Covid-19 Community Mobility Reports [8]. In addition, we have selected four countries initially chosen for evaluation by [23]. These countries are the United States (USA) [43], Britain (UK) [44], Brazil [45], and Russia [46]. We chose the same countries as it would allow us to provide direct comparisons to the results from [23]. Like other Covid-19 research, these countries are the major selection countries because the affected number of people during the pandemic is high, with the most confirmed daily new cases worldwide [42].

Apple’s Mobility Trends Reports [9] provide the movement datasets of people using their transportation, public transportation, and walking. With this dataset, we analyze all possible movements that could be the main factors that would affect the rise of the number of confirmed new cases during the pandemic period. Like the Apple Covid-19 Mobility Trends Reports [9] and Google’s Covid-19 Community Mobility Reports [8] analyze the deadly coronavirus’s spatial transmission that correlates with the daily number of new cases in a particular area. Various data areas have been provided from the dataset, such as retail and recreational areas, grocery and pharmacies, parks, transit stations, workplaces, and residential places. While both Apple’s [9] and Google’s [8] reports are used to capture movement and mobility data, the Our World in Data [42] dataset is used as our basis to correlate both mobility datasets of Apple [9] and Google [8] reports with the number of confirmed people affected by the deadly coronavirus daily.

### 4.2 Data processing

One of the core studies in the analysis is to understand the relationships of future transmissions based on mobility trends. To obtain the value of the relationship, all the datasets must undergo feature extraction. In OWID [42] datasets, the extracted features are daily new cases and dates. These two features are the main core of this analysis to understand the relationships with mobility trends. The daily new cases value pertains to the number of people affected by coronavirus daily.

There are several features from mobility trends datasets being extracted. These features are only shown in the mobility movement and the capacity number of people in activity and public places of a country during a pandemic. However, Apple’s [9] and Google’s [8] datasets differ a bit in terms of features. The extracted features of Apple’s dataset include driving, walking, and transit. The driving feature means the value of people using their vehicles to move in a country. The walking feature means the value people use by walking across the country. Lastly, the transit feature means the value of people using public transportation in a country.

On the other hand, the extracted features from Google’s dataset [8] include retail and recreation, grocery and pharmacy, parks, transit stations, workplaces, and residential. All these features mean the percentage of people moving or attending in specific areas of a country. Therefore, both Apple’s [9] and Google’s [8] datasets have unique and integral features to find logical relationships with the number of new cases daily. Furthermore, all these features will be filtered, combined, and matched to the dates from the OWID’s [42], Apple’s, and Google datasets, respectively, over four selected countries. As a result, each country will have a dataset containing a composition of Apple [9], Google [8], and OWID [42] datasets. These datasets will then be split into two parts of datasets for training and testing purposes. The split will be at 70:30 of training and testing datasets. The final datasets over all four countries will include all the integral features: country name, date, driving, walking, transit, retail and recreation, grocery and pharmacy, parks, transit stations, workplaces, residential, and several new cases daily.

We pre-process the datasets for each country separately. For each country, we will filter, group, and merge Apple’s [9], Google’s [8], and OWID [42] datasets by country name and dates to find the relationship between the movement and mobility with the number of confirmed people affected by the virus. At first, we filter the datasets of Apple’s [9] and Google’s [8] datasets based on four countries as mentioned in subsection 4.1. Then, the data from these datasets will be grouped into dates and country names before merging from Apple’s [9] and Google’s [8] datasets into a single mobility dataset. All of Apple’s and Google’s important features retained from this merging will be analyzed in the latter. To analyze the datasets more effectively, all dataset combinations, including OWID [42] datasets and Apple’s Mobility Trends Reports datasets [9]. Also, Google’s Covid-19 Community Mobility Reports [8] datasets are restructured and divided again into two subsets of the dataset. The first dataset is called March-To-December dataset (MarToDec), structured based on a certain range of dates from 1 March 2020 to 31 December 2020, while the second dataset is called Full dataset, structured based from a range of dates in which all the dataset of countries start collected from 26 February 2020 until the availability of mobility dataset end on 24 March 2022. Both data of the MarToDec and Full datasets will be structured for testing the N-BEATS model into two shots of sets with different splitting range. For the MarToDec dataset, a 1-Shot dataset is encompassed of 70% train subset and 30% test of dataset, while for the 2-Shot dataset is encompassed of half-split (50%) of the MarToDec dataset into series X and 50% for series Y. Both series X and series Y will be half-split again into subsets of training and testing purposes. Therefore, in 2-Shot dataset of the MarToDec dataset will have a set series X dataset that range started from 1 March 2020 to 30 July 2020, while a series Y dataset has a range date started from 31 July 2020 to 31 December 2020. For the Full dataset, the data is also similarly structured into two shots of datasets. The 1-Shot of Full dataset is splitting of 70% of training data and 30% of testing data. On other hand, the 2-Shot of Full dataset will be split with the similar configuration as 2-Shot of dataset from MarToDec dataset. The configuration will generated two series of datasets which called series X and series Y, that ranged from 26 February 2020 to 9 March 2021 for series X, while for series Y ranged from 10 March 2021 to 24 March 2022.

### 4.3 Assessment indicator

Evaluating the model is essential in terms of accuracy and error measurement. Similar statistical evaluation metric has been applied in this analysis to follow the Markov measurement, the Root-Mean-Squared-Error. The Root-Mean-Squared-Error (RMSE) is a standard deviation of residuals used to evaluate the degree of error dispersion. Our focus in this analysis was to make a better model to reduce the error rates from the Markov models. Therefore, in order to capture better insight and evaluation of the N-BEATS model, we have added the MAPE (Mean-Absolute Percentage Error) value for better understanding, and both error rates were defined as the following formulas:


$$\begin{array}{c} RMSE=\sqrt{\frac{1}{n}\sum_{i=1}^{n}{\left(y-\hat{y}\right)}^{2},}\;MAPE=\frac{1}{n}\sum_{i=1}^{n}\frac{\left|A_{i}-F_{i}\right|}{A_{i}} \end{array}$$ (7)

where *y* is the true value and $\hat{y}$ is the predicted value, *n* is the number of values.

Although the reported RMSE and MAPE values were averaged over five runs with different random seeds, formal dependence-aware statistical significance testing was not performed; therefore, the observed improvements should be interpreted as consistent empirical gains rather than confirmed statistical significance.

In the Markov analysis, a similar method has been applied by structuring a model for predicting daily death cases of coronavirus within the date range [23]. Since the analysis began in March and continued through December, the dataset will be referred to as the Mar-To-Dec dataset throughout this analysis.

### 4.4 Interpretability of N-BEATS outputs

While the primary contribution of N-BEATS lies in predictive performance, its architecture also provides interpretability through basis expansion. In our experiments, the model’s trend components aligned with the major pandemic waves, capturing the long-term growth and decline of cases, while the seasonal components reflected shorter cycles such as weekly reporting effects. By integrating mobility covariates, the forecasts further highlight how changes in population movement contributed to epidemic dynamics.

For example, in the USA, the relatively higher RMSE at step 3 in Table 2 reflects abrupt changes in transmission during the early reopening period, when workplace and transit mobility surged and led to sudden increases in predicted cases. In the UK, the drop in error values after step 5 also in Table 2 coincided with the stabilization that followed strict national lockdowns, showing how reductions in mobility were captured by the trend component of the model. In Brazil, the large forecast deviations in Table 4 aligned with the emergence of the Gamma variant, which caused unexpected spikes in cases despite stable mobility patterns. Similarly, in Russia, fluctuations in RMSE across steps in Table 4 were linked to delays in reporting and sudden intervention shifts, which appeared in the seasonal component as short-term volatility.

These examples demonstrate that the N-BEATS forecasts, beyond providing numerical accuracy, can be interpreted as signals of how mobility, government interventions, and variant-driven regime shifts dynamically influenced the spread of COVID-19.

A limitation of this study is that the datasets mainly represent the earlier phases of the COVID-19 pandemic, before vaccination coverage, booster uptake, herd immunity, and variant-specific effects became increasingly important drivers of case dynamics. Consequently, the relationship learned by the model between mobility patterns and daily new cases may be more reliable for early-wave dynamics than for later pandemic stages. In later phases, similar mobility patterns may not correspond to the same transmission intensity because case trends were increasingly shaped by vaccination, waning immunity, emerging variants, testing practices, and changing public-health interventions. Therefore, although the proposed framework performs well on the studied datasets, its generalizability to later-stage pandemic conditions should be interpreted cautiously. Future work should incorporate vaccination-related, immunity-related, variant-related, testing, and policy-intervention variables to improve robustness across evolving epidemic phases.

### 4.5 Experimental results

The proposed models are the final models that have been used to predict the number of daily new cases of Covid-19 in four countries (i.e., the USA, Britain, Brazil, and Russia) using the N-BEATS architecture. To ensure consistency and reproducibility, the N-BEATS model was trained under a fixed set of hyperparameters across all experiments. Table 9 summarizes the key experimental settings, including the lookback window, forecast horizon, network architecture, and optimization strategy. In particular, a 30-day input window was used to predict a 14-day horizon, with each block consisting of four fully connected layers of width 512. Regularization was achieved through a dropout rate of 0.1, and the Adam optimizer with a learning rate of 0.001 was employed for stable convergence. Training was conducted for 200 epochs with a batch size of 64, using Mean Squared Error (MSE) as the loss function. To account for stochastic variability, all experiments were repeated five times with different random seeds, and the reported results correspond to the average across runs.

**Fig 2:**
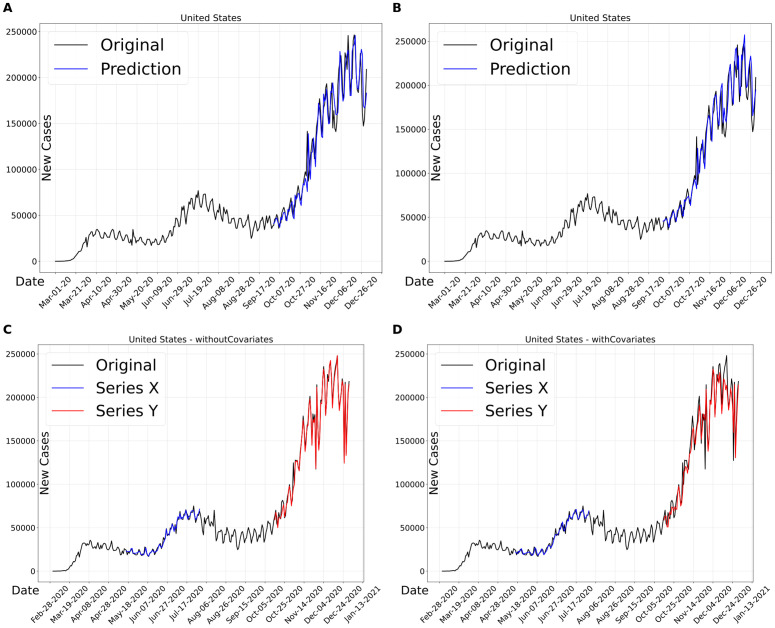
Graph of USA March to December dataset comparison. **(A)** USA without covariates, 1-shot. **(B)** USA with covariates, 1-shot. **(C)** USA without covariates, 2-shot. **(D)** USA with covariates, 2-shot.

**Fig 3:**
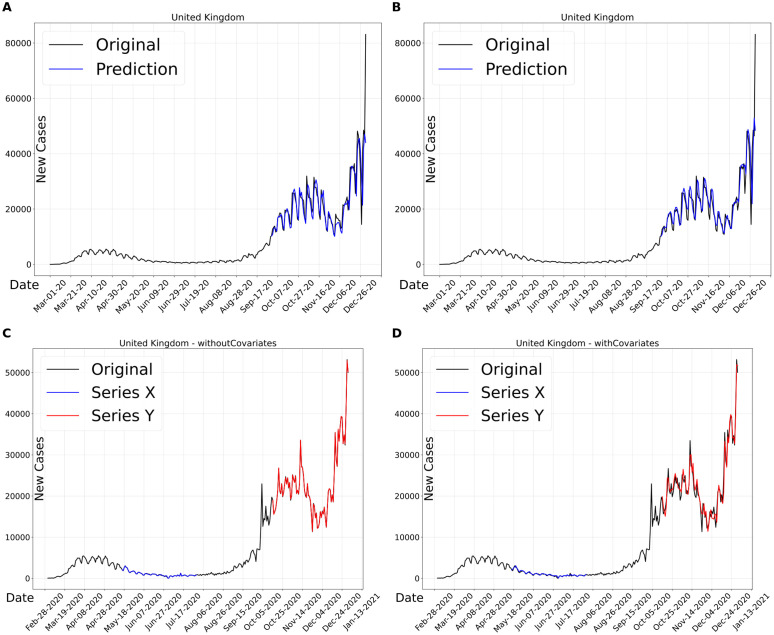
Graph of UK March to December dataset comparison. **(A)** UK without covariates, 1-shot. **(B)** UK with covariates, 1-shot. **(C)** UK without covariates, 2-shot. **(D)** UK with covariates, 2-shot.

**Fig 4:**
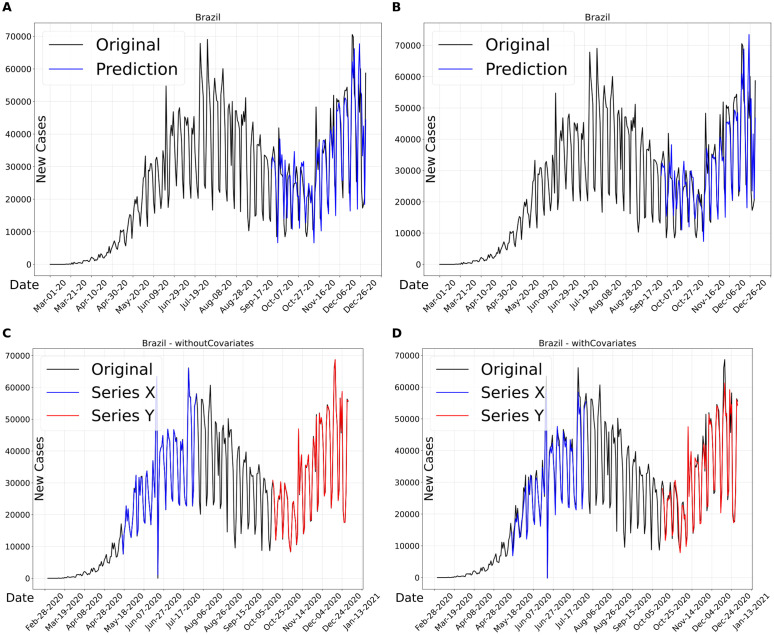
Graph of Brazil March to December dataset comparison. **(A)** Brazil without covariates, 1-shot. **(B)** Brazil with covariates, 1-shot. **(C)** Brazil without covariates, 2-shot. **(D)** Brazil with covariates, 2-shot.

**Fig 5:**
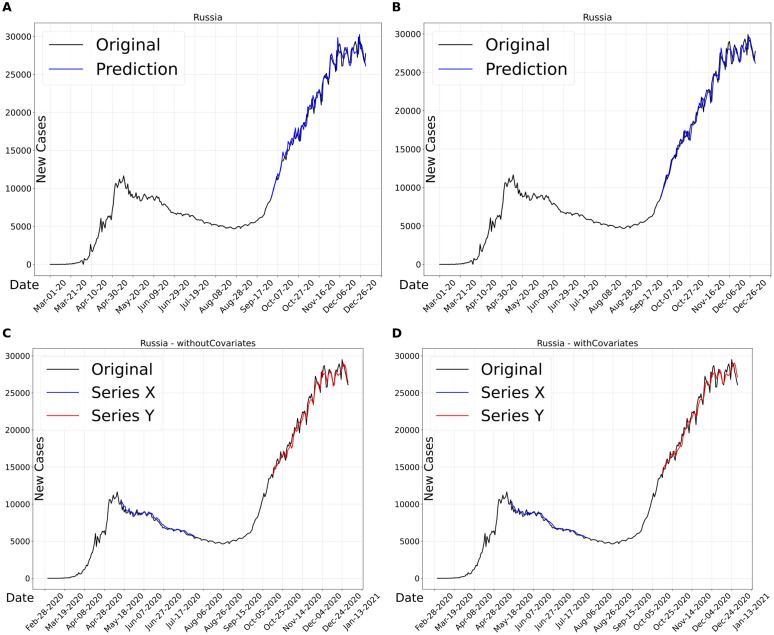
Graph of Russia March to December dataset comparison. **(A)** Russia without covariates, 1-shot. **(B)** Russia with covariates, 1-shot. **(C)** Russia without covariates, 2-shot. **(D)** Russia with covariates, 2-shot.

Figs 2–5 illustrate the comparative impact of covariates on the predictive number of new cases, both with Covariate and without Covariate. While the figures lack a clear delineation of patterns since the N-BEATS models have significantly advanced predictive capabilities, the tables below offer detailed insights into the nuanced relationships between each of without Covariate and with Covariate of the N-BEATS models over the number of new cases across different time steps. The tables above shown the MAPE results of USA and UK in full dataset (Table 1), RMSE results of USA and UK in full dataset (Table 2), MAPE results of Brazil and Russia in full dataset (Table 3), and RMSE results of Brazil and Russia in full dataset (Table 4) composed of both N-BEATS models with covariates and without covariates as well as the models. Tables 5 and 6 are RMSE comparison of using March-To-December dataset in 1-Shot and 2-Shot categories. The experimental result of the N-BEATS models using March-To-December datasets will be compared with the Markov-Analysis model for evaluation in same tables of (Table 5) and (Table 6). All of the N-BEATS models are configured with similar parameters that have shown in the (Table 7). In the (Table 8) shows a comparative cumulative table of the reported new cases and predicted new cases using LSTM-Markov Model and the N-BEATS Model.

**Table 1:** A comparison of the MAPE values for the USA and UK. The MAPE values are calculated for two different models: N-BEATS with Covariate, and N-BEATS without Covariate. The results show that the N-BEATS with Covariate model has the lowest MAPE values for all countries in the table below, indicating its superior forecasting performance.

USA					UK			
1-Shot_(70:30)_		2-Shot${\;}_{X(50:50),Y(50:50)}$		Time	1-Shot_(70:30)_		2-Shot${\;}_{X(50:50),Y(50:50)}$	
withoutCov	withCov	withoutCov	withCov	Step	withoutCov	withCov	withoutCov	withCov
N-BEATS	N-BEATS	N-BEATS	N-BEATS		N-BEATS	N-BEATS	N-BEATS	N-BEATS
3.85E-01	4.00E-01	2.64E-01	2.46E-01	3	1.41E-01	1.08E-01	4.52E + 18	4.41E + 18
4.40E-01	3.84E-01	1.82E-01	1.72E-01	4	1.06E-01	1.36E-01	2.90E + 18	2.83E + 18
4.39E-01	3.88E-01	1.59E-01	2.00E-01	5	1.08E-01	1.30E-01	1.64E + 18	1.32E + 18
3.30E-01	3.70E-01	1.51E-01	1.75E-01	6	1.05E-01	8.47E-02	1.52E + 18	1.56E + 18
5.63E-01	3.71E-01	1.09E-01	1.25E-01	7	9.77E-02	8.22E-02	7.42E + 17	1.10E + 18
3.14E-01	3.42E-01	1.66E-01	1.11E-01	8	**7.61E-02**	7.60E-02	9.06E + 17	8.90E + 17
4.32E + 00	**2.50E-01**	8.80E-02	1.36E-01	9	7.83E-02	7.40E-02	3.72E + 17	9.51E + 17
3.56E-01	2.91E-01	9.91E-02	1.14E-01	10	8.52E-02	8.29E-02	1.19E + 18	1.05E + 18
2.81E-01	2.69E-01	7.98E-02	1.24E-01	11	8.00E-02	**7.26E-02**	2.38E + 17	9.16E + 17
3.40E-01	3.06E-01	1.37E-01	1.27E-01	12	7.90E-02	7.56E-02	1.22E + 18	9.40E + 17
3.70E-01	3.20E-01	8.38E-02	1.54E-01	13	7.92E-02	7.81E-02	8.49E + 17	1.74E + 18
3.20E-01	3.04E-01	**7.53E-02**	**1.02E-01**	14	8.85E-02	9.23E-02	1.05E + 18	9.47E + 17
**2.68E-01**	3.82E-01	7.87E-02	1.28E-01	15	9.46E-02	9.92E-02	**2.22E + 17**	**8.55E + 17**

**Table 2:** A comparison of the RMSE values for the USA and UK. The RMSE values are calculated for two different models: N-BEATS with Covariate, and N-BEATS without Covariate. The results show that the N-BEATS with Covariate model has the lowest RMSE values for all countries in the table below, indicating its superior forecasting performance.

USA					UK			
1-Shot_(70:30)_		2-Shot${\;}_{X(50:50),Y(50:50)}$		Time	1-Shot_(70:30)_		2-Shot${\;}_{X(50:50),Y(50:50)}$	
withoutCov	withCov	withoutCov	withCov	Step	withoutCov	withCov	withoutCov	withCov
N-BEATS	N-BEATS	N-BEATS	N-BEATS		N-BEATS	N-BEATS	N-BEATS	N-BEATS
112,239	116,348	66,584	59,597	3	15,564	16,575	12,752	12,518
145,828	118,504	39,645	51,591	4	14,232	15,362	11,001	10,772
125,892	119,819	39,364	41,860	5	14,310	15,074	10,821	9,947
106,936	111,682	33,676	54,101	6	16,500	12,371	7,305	8,500
306,926	116,196	19,097	23,139	7	14,140	12,673	5,927	7,547
100,784	93,615	26,072	15,863	8	11,050	12,803	5,961	6,354
5,747,075	**81,689**	14,688	40,896	9	12,108	12,547	2,871	**5,242**
147,381	88,650	15,708	23,251	10	12,755	12,480	4,548	5,918
90,539	85,950	12,148	28,544	11	**9,394**	10,884	2,979	6,836
97,158	90,505	45,985	31,054	12	9,488	10,705	5,979	6,390
105,484	90,793	12,076	38,154	13	10,886	10,740	6,372	12,105
88,789	82,737	**11,801**	**14,568**	14	10,489	11,721	5,535	5,475
**81,232**	96,593	12,713	28,023	15	10,969	**10,460**	**2,332**	7,405

**Table 3:** A comparison of the MAPE values for the Brazil and Russia. The MAPE values are calculated for two different models: N-BEATS with Covariate, and N-BEATS without Covariate. The results show that the N-BEATS with Covariate model has the lowest MAPE values for all countries in the table below, indicating its superior forecasting performance.

Brazil					Russia			
1-Shot_(70:30)_		2-Shot${\;}_{X(50:50),Y(50:50)}$		Time	1-Shot_(70:30)_		2-Shot${\;}_{X(50:50),Y(50:50)}$	
withoutCov	withCov	withoutCov	withCov	Step	withoutCov	withCov	withoutCov	withCov
N-BEATS	N-BEATS	N-BEATS	N-BEATS		N-BEATS	N-BEATS	N-BEATS	N-BEATS
1.68E + 18	**1.59E + 18**	3.93E + 17	3.67E + 17	3	**4.02E-02**	**4.49E-02**	4.12E-02	3.65E-02
2.07E + 18	2.00E + 18	3.63E + 17	3.52E + 17	4	4.71E-02	5.16E-02	3.79E-02	4.18E-02
1.99E + 18	1.85E + 18	3.53E + 17	3.15E + 17	5	4.82E-02	4.87E-02	4.26E-02	4.71E-02
2.22E + 18	1.88E + 18	1.90E + 17	1.89E + 17	6	4.76E-02	8.44E-02	3.39E-02	3.62E-02
2.01E + 18	1.89E + 18	2.02E + 17	1.16E + 17	7	5.58E-02	4.62E-02	3.43E-02	3.18E-02
2.06E + 18	1.83E + 18	4.58E + 15	4.49E + 16	8	5.54E-02	6.31E-02	4.11E-02	4.70E-02
2.09E + 18	2.11E + 18	1.10E + 16	1.64E + 16	9	5.55E-02	4.66E-02	3.04E-02	3.33E-02
**1.44E + 18**	1.94E + 18	4.18E + 16	3.03E + 16	10	5.49E-02	5.93E-02	**2.48E-02**	3.51E-02
1.79E + 18	2.00E + 18	1.30E + 16	4.08E + 16	11	4.56E-02	5.76E-02	2.75E-02	3.56E-02
1.69E + 18	1.81E + 18	7.28E + 15	1.09E + 17	12	7.70E-02	6.56E-02	2.50E-02	3.61E-02
1.90E + 18	2.23E + 18	**3.48E + 15**	**3.99E + 15**	13	6.58E-02	6.13E-02	3.79E-02	5.96E-02
2.17E + 18	2.28E + 18	1.42E + 16	1.32E + 17	14	6.99E-02	7.20E-02	3.70E-02	**2.88E-02**
2.45E + 18	2.51E + 18	9.02E + 15	2.09E + 17	15	6.81E-02	6.22E-02	2.55E-02	8.10E-02

**Table 4:** A comparison of the RMSE values for the Brazil and Russia. The RMSE values are calculated for two different models: N-BEATS with Covariate, and N-BEATS without Covariate. The results show that the N-BEATS with Covariate model has the lowest RMSE values for all countries in the table below, indicating its superior forecasting performance.

Brazil					Russia			
1-Shot_(70:30)_		2-Shot${\;}_{X(50:50),Y(50:50)}$		Time	1-Shot_(70:30)_		2-Shot${\;}_{X(50:50),Y(50:50)}$	
withoutCov	withCov	withoutCov	withCov	Step	withoutCov	withCov	withoutCov	withCov
N-BEATS	N-BEATS	N-BEATS	N-BEATS		N-BEATS	N-BEATS	N-BEATS	N-BEATS
43,077	32,332	14,694	15,135	3	**4,527**	5,078	3,226	2,945
33,662	34,114	9,375	9,827	4	4,877	5,200	2,941	3,438
33,259	32,436	9,413	8,695	5	5,069	**4,970**	2,847	4,233
29,435	28,706	7,526	5,903	6	4,938	7,372	2,754	2,955
32,913	26,475	11,201	5,193	7	6,004	5,067	2,383	2,279
28,102	30,472	4,717	5,193	8	6,311	6,351	3,450	2,346
29,135	31,650	2,837	4,531	9	6,166	5,312	1,835	**1,636**
27,819	28,217	3,530	8,299	10	6,788	5,483	1,028	3,286
26,316	28,257	2,845	7,874	11	5,728	5,667	1,462	2,006
**25,318**	27,716	**1,679**	6,119	12	6,321	7,140	**999**	1,832
28,324	**24,978**	2,424	**4,278**	13	6,930	6,784	2,287	2,417
26,279	25,214	2,038	9,900	14	7,469	7,524	1,355	2,077
27,724	27,596	1,778	5,556	15	7,067	6,752	1,264	6,347

**Table 5:** A comparison of the RMSE values for the USA and UK. The RMSE values are calculated for two different models: N-BEATS with Covariate, and N-BEATS without Covariate. The results show that the N-BEATS with Covariate model has the lowest RMSE values for all countries in the table below, indicating its superior forecasting performance.

USA						
Time	Ma. et. al.		1-Shot_(70:30)_		2-Shot${\;}_{X(50:50),Y(50:50)}$	
Step	LSTM	LSTM	withoutCov	withCov	withoutCov	withCov
		Markov	N-BEATS	N-BEATS	N-BEATS	N-BEATS
3	3,271,974	2,204,629	21,163	**16,580**	10,609	12,644
4	4,298,475	1,818,030	17,622	17,491	14,280	10,671
5	4,298,801	2,755,216	17,321	17,181	11,190	12,430
6	4,533,149	2,636,602	17,648	17,961	11,540	12,145
7	4,659,424	2,957,439	**16,868**	16,597	**3,758**	15,784
8	4,465,570	3,037,668	16,990	17,346	4,169	13,444
9	**3,006,406**	**1,249,420**	18,543	16,649	7,695	**9,814**
10	3,418,221	2,920,985	18,664	16,620	3,943	10,176
11	5,026,319	2,632,837	20,601	17,873	8,633	11,553
12	4,912,377	4,251,474	17,560	17,340	7,470	10,412
13	4,501,122	2,717,536	18,171	17,044	6,047	11,503
14	4,365,950	2,889,534	17,716	18,054	14,200	14,779
15	3,773,912	3,775,956	18,495	17,439	8,433	12,630
**UK**						
**Time**	**Ma. et. al.**		**1-Shot**_**(70:30)**_		2-Shot${\;}_{X(50:50),Y(50:50)}$	
**Step**	**LSTM**	**LSTM**	**withoutCov**	**withCov**	**withoutCov**	**withCov**
		**Markov**	**N-BEATS**	**N-BEATS**	**N-BEATS**	**N-BEATS**
3	426,583	248,493	6,569	5,893	2,462	2,454
4	239,493	485,919	6,592	6,559	2,015	3,000
5	421,336	692,762	6,802	5,995	2,062	2,276
6	**219,766**	306,029	6,044	**5,611**	756	1,970
7	331,109	**236,511**	6,086	5,968	744	1,630
8	344,832	579,751	5,851	5,720	948	1,489
9	440,334	244,718	5,791	6,554	524	**1,205**
10	358,119	754,001	5,984	6,521	569	1,226
11	715,564	257,792	5,804	5,920	135	1,506
12	410,471	380,434	6,118	6,335	764	3,057
13	985,517	258,268	5,841	5,975	299	1,294
14	468,084	305,483	5,878	5,908	**97**	1,347
15	526,129	1,314,939	**5,704**	6,516	184	2,449

**Table 6:** A comparison of the RMSE values for the Brazil and Russia. The RMSE values are calculated for two different models: N-BEATS with Covariate, and N-BEATS without Covariate. The results show that the N-BEATS with Covariate model has the lowest RMSE values for all countries in the table below, indicating its superior forecasting performance.

Brazil						
Time	Ma. et. al.		1-Shot_(70:30)_		2-Shot${\;}_{X(50:50),Y(50:50)}$	
Step	LSTM	LSTM	withoutCov	withCov	withoutCov	withCov
		Markov	N-BEATS	N-BEATS	N-BEATS	N-BEATS
3	698,082	506,549	11,029	11,209	7,846	8,415
4	807,812	422,905	12,790	12,425	3,225	5,366
5	1,028,703	590,634	9,088	9,936	5,308	3,888
6	884,107	333,420	**8,259**	**8,749**	3,109	3,981
7	1,105,731	431,629	8,835	10,227	1,125	2,619
8	726,134	245,414	9,091	9,034	1,256	4,615
9	760,957	379,625	9,452	8,908	2,640	6,459
10	**432,045**	**199,163**	9,660	8,999	336	3,775
11	742,365	555,268	10,042	10,053	**303**	2,879
12	1,072,279	723,964	10,241	9,975	496	2,870
13	906,820	380,523	9,379	9,795	672	**2,583**
14	1,033,211	414,927	9,252	9,240	329	4,260
15	953,686	322,795	8,967	9,618	382	3,828
**Russia**						
**Time**	**Ma. et. al.**		**1-Shot**_**(70:30)**_		2-Shot${\;}_{X(50:50),Y(50:50)}$	
**Step**	**LSTM**	**LSTM**	**withoutCov**	**withCov**	**withoutCov**	**withCov**
		**Markov**	**N-BEATS**	**N-BEATS**	**N-BEATS**	**N-BEATS**
3	586,483	139,460	927	1,060	808	860
4	381,164	139,842	1,072	912	717	1,074
5	169,464	246,306	1,024	1,239	683	905
6	532,194	210,934	1,408	1,674	651	688
7	440,670	**89,942**	1,421	948	683	1,142
8	464,402	338,961	1,249	1,222	665	1,229
9	476,284	252,601	1,042	1,053	618	805
10	303,172	238,583	**915**	989	**550**	845
11	369,349	327,625	995	1,421	690	849
12	245,291	312,600	994	922	566	**607**
13	520,249	140,814	993	942	567	741
14	**76,835**	143,322	1,300	**859**	729	1,259
15	285,784	198,222	919	1,083	737	1,310

**Table 7:** Final model parameters.

Country	Layer	Dataset	Model	Dropout	Epochs	Stacks	Blocks	Widths	Coeff.	Deg.
US	4	Mar-To-Dec	N-BEATS without covariates	NA	100	30	1	256	5	2
	4	Mar-To-Dec	N-BEATS with covariates	NA	100	30	1	256	5	2
Britain	4	Mar-To-Dec	N-BEATS without covariates	NA	100	30	1	256	5	2
	4	Mar-To-Dec	N-BEATS with covariates	NA	100	30	1	256	5	2
Brazil	4	Mar-To-Dec	N-BEATS without covariates	NA	100	30	1	256	5	2
	4	Mar-To-Dec	N-BEATS with covariates	NA	100	30	1	256	5	2
Russia	4	Mar-To-Dec	N-BEATS without covariates	NA	100	30	1	256	5	2
	4	Mar-To-Dec	N-BEATS with covariates	NA	100	30	1	256	5	2

**Table 8:** Comparison of cumulative confirmed cases reported and predicted daily.

Country	Date	Reported value	LSTM-Markov	LSTM-Markov	N-BEATS	N-BEATS	N-BEATS	N-BEATS
				error rate	without covariate	error rate	with covariate	error rate
US	2020.12.5	14,697,256	14,564,888	0.0091	14,757,534	0.0041	14,331,512	0.0255
	2021.1.5	21,264,846	20,506,525	0.0370	21,656,791	0.0181	20,795,978	0.0225
	2021.2.5	26,961,530	24,782,471	0.0879	27,857,651	0.0322	26,706,427	0.0096
Britain	2020.12.5	1,707,433	1,492,824	0.1438	1,721,481	0.0082	1,675,875	0.0188
	2021.1.5	2,777,606	2,773,468	0.0015	2,759,713	0.0065	2,664,303	0.0425
	2021.2.5	3,915,414	3,883,746	0.0082	3,963,558	0.0121	3,813,562	0.0267
Brazil	2020.12.5	6,600,313	6,271,946	0.0524	6,799,287	0.0293	6,556,303	0.0067
	2021.1.5	7,839,432	7,625,120	0.0281	8,192,032	0.0430	7,858,429	0.0024
	2021.2.5	9,476,296	9,191,593	0.0310	9,812,395	0.0343	9,430,843	0.0048
Russia	2020.12.5	2,410,462	2,576,114	0.0643	2,351,881	0.0249	2,347,623	0.0268
	2021.1.5	3,250,713	3,162,229	0.0280	3,184,125	0.0210	3,178,867	0.0226
	2021.2.5	3,891,274	3,638,792	0.0694	3,824,859	0.0174	3,819,141	0.0189

**Table 9:** Experimental settings for N-BEATS training. Each experiment was repeated five times with different random seeds, and average results are reported.

Parameter	Value
Lookback window (input chunk length)	30 days
Forecast horizon	14 days
Blocks per stack	4
Layers per block	4 fully connected layers
Layer width	512
Expansion coefficient dimension	32
Trend polynomial degree	2
Dropout rate	0.1
Optimizer	Adam
Learning rate	0.001
Batch size	64
Epochs	200
Loss function	Mean Squared Error (MSE)
Repetitions	5 runs (averaged)

Both N-BEATS models had been trained using the OWID dataset to predict new confirmed cases daily among people in the country. However, only the N-BEATS with covariate was supported with the additional features of Apple and Google mobility report datasets. The intention of additional features in the N-BEATS with covariates was to identify whether the reports would help reduce the error rates and improve the accuracy by including mobility as covariates for the model. Initially, we established the range of input time steps. Then, through trial and error, we identified the optimal window value and allocated each country with its corresponding best time step.

#### 4.5.1 USA analysis.

In the 1-Shot category from the Tables 1 and 2, which involve making predictions based on a single input, both N-BEATS without Covariate and N-BEATS with Covariate achieve their best MAPE and RMSE values at different time-steps. Specifically, N-BEATS without Covariate achieves a MAPE of 2.68E-01 and RMSE of 81,232. Both occurs at time-step 15. While N-BEATS with Covariate achieves a slightly better MAPE of 2.50E-01 at time-step 9 and 81,689 in RMSE at similar time-step. The N-BEATS without Covariate has produced the worst result in the highest-case scenarios. It has produced a MAPE and RMSE results of 4.32E + 00 and 5,747,075 at the same time-step of 9.

Transitioning to the 2-Shot category, where forecasts are made based on two consecutive inputs on different parts of dates, N-BEATS without Covariate outperforms N-BEATS with Covariate in terms of the lowest possible MAPE and RMSE value. Specifically, N-BEATS without Covariate achieves a lowest possible MAPE of 7.53E-02 at time-step 14, while N-BEATS with Covariate achieves a MAPE of 1.02E-01 at the same time-step. Similar case happen in the RMSE, the N-BEATS without Covariate achieved slightly better with 11,801–14,568 of the N-BEATS with Covariate at the same time-step of 14. Both MAPE and RMSE were the best results in the USA analysis. In the highest scenario, the N-BEATS without Covariate demonstrating the highest MAPE of 2.64E-01 at time-step 3, compared to 2.46E-01 at the same time-step for N-BEATS with Covariate in this 2-Shot category at similar time-step. Similar to the RMSE, both N-BEATS with Covariate and without Covariate received 66,584 and 59,597 at the same time-step.

#### 4.5.2 UK analysis.

In the UK’s 1-Shot category from the Tables 1 and 2, where predictions are based on a single input, both N-BEATS models achieve their best MAPE and RMSE values at different time steps. Specifically, the N-BEATS with Covariate attains a best MAPE of 7.26E-02 at time-step 11, while for the best RMSE was produced from N-BEATS without Covariate of 9,394 at time-step 11. However, when assessing highest MAPE and RMSE, the N-BEATS without Covariate reporting a worst MAPE of 1.41E-01 at time-step 3, while for the worst RMSE was generated by N-BEATS with Covariate of 16,575 at time-step 3, closely followed by 16,500 at time-step 6 for the N-BEATS without Covariate.

Transitioning to the 2-Shot category, where forecasts are made based on two consecutive inputs, the N-BEATS without Covariate has performed the best MAPE and RMSE values. Both have achieve the best MAPE and RMSE values of 2.22E + 17 and 2,332 respectively at the same time-step of 15. However, in the worst-case scenarios, with the N-BEATS model has demonstrated extremely high MAPE and RMSE values. The N-BEATS without Covariate showing a the highest MAPE and RMSE of 4.52E + 18 and 12,752 at the same time-step of 3, followed closedly by the N-BEATS with Covariate of 4.41E + 18 MAPE and 12,518 RMSE at the same time-step of 3.

It is worth noting that the very large MAPE values is because MAPE normalizes the absolute error by the ground‑truth value. When daily case counts are very small (e.g., early outbreak periods or brief low‑count intervals), the denominator becomes small and the percentage error can become disproportionately large. Accordingly, MAPE is interpreted primarily for within‑dataset model comparison, while RMSE is emphasized as a more stable indicator of absolute predictive accuracy in low‑count regimes.

#### 4.5.3 Brazil analysis.

In the 1-Shot category from the Tables 3 and 4, where predictions are based on a single input, N-BEATS without Covariate achieves the best MAPE with a slightly lower of lowest MAPE value of 1.44E + 18 at time-step 8, compared to 1.59E + 18 at time-step 11 for the lowest MAPE of the N-BEATS with Covariate in the 1-Shot category. However, the difference in performance is relatively small. In the RMSE metrics, the N-BEATS with Covariate has exhibited the lowest of the 1-Shot RMSE value, achieving 24,978 at time-step 13, closedly followed by the N-BEATS without Covariate with 25,318 at time-step 12. When considering the highest value of MAPE scenario, with the N-BEATS with Covariate yielding a worst MAPE of 2.51E + 18 at time-step 15, also closely followed by 2.51E + 18 at the same time-step for the N-BEATS without Covariate. However, in the highest value of RMSE scenario, the N-BEATS without Covariate has exhibited the worst value in the Brazil’s RMSE metrics, produced of 43,077 RMSE value at time-step 3.

Transitioning to the 2-Shot category, the N-BEATS without Covariate again demonstrates slightly better performance than the N-BEATS with Covariate, achieving a best MAPE value of 3.48E + 15 at time-step 13, compared to 3.99E + 15 at the same time-step for N-BEATS with Covariate. Similar case in RMSE, the N-BEATS without Covariate has achieved better performance with 1,679 at time-step 12 of RMSE compared to 4,278 at time-step 13. When examining the highest of MAPE and RMSE values, the difference in performance narrows, with both models showing high MAPE values, but N-BEATS without Covariate exhibiting a slightly lower MAPE of 3.67E + 17 and RMSE of 14,694 at time-step 3, compared to 3.93E + 17 MAPE and 15,135 RMSE at the same time-step for N-BEATS with Covariate.

#### 4.5.4 Russia analysis.

In the Russia’s 1-Shot category from the Tables 3 and 4, both models have exhibited the best MAPE values at the same time-step of 3. The N-BEATS without Covariate has slightly performed better MAPE value with 4.02E-02, while the N-BEATS with Covariate with 4.49E-02. In the RMSE metrics, both N-BEATS models achieve their best RMSE values at different time steps. N-BEATS without Covariate demonstrates a best RMSE of 4,527 at time-step 8, while N-BEATS with Covariate slightly fall behind this with a RMSE of 4,970 at time-step 11. However, in highest MAPE and RMSE scenarios, both models exhibit comparable performance, with N-BEATS with Covariate yielding the highest MAPE of 8.44E-02 at time-step 6, closely followed by 7.70E-02 at time-step 12 for N-BEATS without Covariate. Both models in highest RMSE exhibit comparable performance, with N-BEATS without Covariate reporting a lesser RMSE of 7,469 at time-step 14, closely followed by 7,524 at the same time-step for N-BEATS with Covariate.

Transitioning to the 2-Shot category, the N-BEATS without Covariate has slightly outperformed the N-BEATS with Covariate in both metrics errors, achieving the best MAPE and RMSE values. Both record the best MAPE of 2.48E-02 and RMSE of 999 at time-step 10 and 15 respectively. However, in the highest error scenario, the performance difference becomes more pronounced, with N-BEATS without Covariate reporting a slightly lower MAPE of 4.26E-02 at time-step 5 and RMSE of 3,450 at time-step 8, compared to 8.10E-02 MAPE and 6,347 RMSE at the same time-step of 15 from the N-BEATS with Covariate.

#### 4.5.5 USA and UK RMSE analysis direct comparison with Markov-analysis.

On the Table 5 shows the analysis of the USA March-to-December dataset reveals intriguing insights into the forecasting performance of N-BEATS models across both the 1-Shot and 2-Shot categories.

In the 1-Shot category, N-BEATS without Covariate achieved its best RMSE value of 16,868 at time-step 7, while N-BEATS with Covariate performed slightly better with a RMSE of 16,580 at time-step 3. However, delving into worst-case scenarios, N-BEATS without Covariate recorded a higher RMSE of 21,163 at time-step 3, compared to 18,054 at time-step 14 for N-BEATS with Covariate.

Transitioning to the 2-Shot category, N-BEATS without Covariate outperformed N-BEATS with Covariate, achieving a best RMSE value of 3,758 at time-step 7, while N-BEATS with Covariate reported a RMSE of 9,814 at time-step 9. Conversely, in worst-case scenarios, N-BEATS without Covariate exhibited a higher RMSE of 14,280 at time-step 4, compared to 15,784 at time-step 7 for N-BEATS with Covariate.

Comparing the USA N-BEATS models with LSTM-Markov highlights substantial differences in RMSE values. In the 1-Shot category, the RMSE of LSTM-Markov differs significantly from N-BEATS without Covariate and with Covariate, with variations of 194.67% and 194.76%, respectively. Similarly, in the 2-Shot category, the RMSE differences between LSTM-Markov and N-BEATS without Covariate and with Covariate are substantial, with variations of 198.8% and 196.88%, respectively. These comparisons underscore the distinct predictive capabilities of N-BEATS models compared to LSTM-Markov in capturing temporal dependencies and forecasting accuracy in the USA dataset.

Analyzing the March-to-December dataset for the UK provides valuable insights into the forecasting performance of N-BEATS models in both the 1-Shot and 2-Shot categories. On the Table 5 shows the Markov-Analysis to be compared with the N-BEATS of 1-Shot and 2-Shot.

In the 1-Shot category, N-BEATS without Covariate achieved its best RMSE value of 5,704 at time-step 8, while N-BEATS with Covariate performed slightly better with a RMSE of 5,611 at time-step 6. However, in terms of worst-case scenarios, N-BEATS without Covariate recorded a higher RMSE of 6,802 at time-step 5, compared to 6,559 at time-step 4 for N-BEATS with Covariate.

Transitioning to the 2-Shot category, N-BEATS without Covariate outperformed N-BEATS with Covariate, achieving a best RMSE value of 97 at time-step 14, while N-BEATS with Covariate reported a RMSE of 1,205 at time-step 9. However, in the worst-case scenarios, N-BEATS without Covariate exhibited a higher RMSE of 2,462 at time-step 3, compared to 3,057 at time-step 12 for N-BEATS with Covariate.

Comparing N-BEATS models with LSTM-Markov reveals significant differences in RMSE values. In the 1-Shot category, the RMSE of LSTM-Markov differs substantially from N-BEATS without Covariate and with Covariate, with variations of 190.58% and 190.73%, respectively. Similarly, in the 2-Shot category, the RMSE differences between LSTM-Markov and N-BEATS without Covariate and with Covariate are substantial, with variations of 199.84% and 197.97%, respectively. These comparisons highlight the distinct predictive capabilities of N-BEATS models compared to LSTM-Markov in capturing temporal dependencies and forecasting accuracy in the UK dataset.

#### 4.5.6 Brazil and Russia RMSE Analysis direct comparison with Markov-analysis.

Examining the March-to-December dataset on the Table 6 for Brazil sheds light on the predictive performance of N-BEATS models across both the 1-Shot and 2-Shot categories.

In the 1-Shot category, both N-BEATS without Covariate and N-BEATS with Covariate achieved their best RMSE values at time-step 6, with 8,259 and 8,749, respectively. However, when considering worst-case scenarios, both models demonstrated comparable performance, with RMSE values of 12,790 and 12,425 at time-step 4 for N-BEATS without Covariate and with Covariate, respectively.

Transitioning to the 2-Shot category, N-BEATS without Covariate performed significantly better, reporting a best RMSE value of 303 at time-step 11, compared to 2,583 at time-step 13 for N-BEATS with Covariate. Similarly, in worst-case scenarios, N-BEATS without Covariate exhibited a lower RMSE of 7,846 at time-step 3, compared to 8,415 at the same time-step for N-BEATS with Covariate.

Comparing the performance of N-BEATS models with LSTM-Markov reveals substantial differences in RMSE values. In both the 1-Shot and 2-Shot categories, the RMSE of LSTM-Markov differs significantly from N-BEATS without Covariate and with Covariate, with variations of 184.07% and 183.17%, respectively, in the 1-Shot category, and 199.39% and 194.88%, respectively, in the 2-Shot category. These comparisons underscore the distinct predictive capabilities of N-BEATS models compared to LSTM-Markov in capturing temporal dependencies and forecasting accuracy in the Brazil dataset.

Analyzing from the Table 6 of the March-to-December dataset for Russia provides insights into the forecasting performance of N-BEATS models in both the 1-Shot and 2-Shot categories.

In the 1-Shot category, N-BEATS without Covariate achieved its best RMSE value of 915 at time-step 10, slightly outperforming N-BEATS with Covariate, which recorded a best RMSE of 859 at time-step 14. However, in worst-case scenarios, N-BEATS without Covariate reported a higher RMSE of 1,421 at time-step 7, compared to 1,674 at time-step 6 for N-BEATS with Covariate.

Transitioning to the 2-Shot category, N-BEATS without Covariate performed better, reporting a best RMSE value of 550 at time-step 10, while N-BEATS with Covariate achieved a best RMSE of 607 at time-step 12. Conversely, in worst-case scenarios, N-BEATS without Covariate exhibited a lower RMSE of 808 at time-step 3, compared to 1,310 at time-step 15 for N-BEATS with Covariate.

Comparing the performance of N-BEATS models with LSTM-Markov highlights significant differences in RMSE values. In both the 1-Shot and 2-Shot categories, the RMSE of LSTM-Markov differs notably from N-BEATS without Covariate and with Covariate, with variations of 195.97% and 196.22%, respectively, in the 1-Shot category, and 197.57% and 197.32%, respectively, in the 2-Shot category. These comparisons underscore the distinct predictive capabilities of N-BEATS models compared to LSTM-Markov in capturing temporal dependencies and forecasting accuracy in the Russia dataset.

## 5 Conclusion

In this paper, we have applied the principles of deep learning to forecast daily COVID-19 new cases. We have utilized a unique neural architecture known as N-BEATS. This architecture is characterized by the presence of backward and forward residual links and a deep stack of fully-connected layers. Remarkably, this architecture can handle long input sequences and large output horizons without losing information or increasing computational complexity. LSTM-Markov, in contrast, suffers from the vanishing gradient problem and requires more memory and time to process long sequences, which reduces their prediction accuracies.

Our study focused on four major countries: the United States, Russia, the United Kingdom, and Brazil. We evaluated the performance of N-BEATS on these countries using three distinct COVID-19 datasets provided by Google, Apple, and OWID. The performance of N-BEATS was compared with that of the LSTM-Markov model, a state-of-the-art model in this domain.

Our results were highly encouraging. N-BEATS demonstrated superior performance, consistently yielding lower Root Mean Squared Error (RMSE) values across all evaluated datasets. This indicates that N-BEATS was able to predict the daily COVID-19 new cases with a higher degree of accuracy compared to the LSTM-Markov model. Furthermore, we found that the accuracy of N-BEATS was further enhanced when we incorporated mobility data from Google and Apple as covariates. This not only improved the accuracy of the predictions but also increased the interpretability of the model, providing more meaningful insights into the factors that influencing the spread of COVID-19. Our work has demonstrated the effectiveness of deep learning, and specifically the N-BEATS architecture, for forecasting COVID-19 new cases. It has also highlighted the value of incorporating mobility data into the model. These findings provide valuable insights for policymakers and public health officials, helping them make more informed decisions in managing the pandemic.

In conclusion, both configurations of N-BEATS, with and without covariates, outperformed the LSTM-Markov model. However, the N-BEATS model with covariates demonstrated better performance than the N-BEATS model without covariates. The N-BEATS models consistntly yielded lower RMSE scores for almost all statistical measures in all four countries’ datasets applied in the experiment. The reduction in RMSE scores of the N-BEATS models, signified the superiority of the model and its adaptability with the environment as the range number of dates increased. Most of the N-BEATS graphs have a prediction line closely adhered to the new cases line, signifying the superiority of the N-BEATS model compared to the Markov model in the Markov analysis. Most of the N-BEATS with covariates graphs closely follow the new case lines. This research has shown that mobility data can provide good predictive power to produce accurate predictions on COVID-19 transmissions, which may aid policymakers in an upcoming pandemic.

The deployment of mobility dataset analysis in evaluating government interventions aimed at curbing disease spread is crucial. Several positive implications can be achieved, including the provision of timely information about the effects of recent interventions, which supports operational policy decisions. Through these operational policies, the government can intervene by implementing regional traffic management strategies to reduce congestion, particularly in severely affected areas. Following such interventions, preemptive awareness campaigns and public guidelines can be provided to promote effective mitigation of disease transmission.

According to [47], human mobility analysis is strongly associated with regional socioeconomic indicators, such as income and poverty rates, and the relationship between mobility and socioeconomic status may vary across cities. These associations are largely influenced by the spatial arrangement of housing, employment opportunities, and human activities. However, this relationship remains a potential subject for further investigation.

In real-time implementation, the accuracy and interpretability of mobility-based COVID-19 forecasts depend strongly on the stipulation of clear government policies. In accordance with [48], since population movement responds directly to public health interventions, transparent and timely communication of policy measures (e.g., mobility restrictions, reopening phases) allows the forecasting model to recalibrate appropriately. Conversely, inconsistent or delayed policy announcements may distort mobility patterns and reduce predictive stability.
