# Supplementary material for: Forecasting COVID-19 new cases using NBEATS deep learning and mobility data
Source: PLoS One. 2026 Jun 29;21(6):e0350264. doi: 10.1371/journal.pone.0350264 (PMC13313360; doi:10.1371/journal.pone.0350264)
Supplement: Attachment — Submitted filename: Binder1.pdf [file pone.0350264.s001.pdf]

Original Manuscript ID: PONE-D-23-17624R3

Original Article Title: "Forecasting COVID-19 New Cases Using NBEATS Deep Learning and Mobility Data"

To: PLOS ONE Editor

Re: Response to reviewers

Dear Editorial Team,

Thank you for the opportunity to resubmit our manuscript. You have requested a point-by-point response to the original reviewers' comments from PONE-D-23-17624R3. Please note:

All original reviewer comments were fully addressed in our revised submission, which was formally **accepted on 19 September 2024**.
No further scientific or analytical changes have been made to the manuscript since that acceptance.
The **only revision** in this new submission is an update of the corresponding-author designation (to Dr Abdul-Halim Jallad) on the title page and metadata.

I have included as an attachment to this letter, the latest correspondence that was received from the academic editor of PLOS ONE verifying that all the reviewers have accepted the final version of the manuscript. Because the manuscript as accepted on 19 September 2024 already incorporates **all** peer-review changes, and no new scientific edits have been made beyond the corresponding-author update, we respectfully request that this resubmission be treated as an **administrative revision** and **not** sent for further external peer review.

Thank you for your consideration. We remain at your disposal if any additional information is needed.

Sincerely,

Abdul-Halim Jallad
Associate Professor, Department of Electrical Engineering
United Arab Emirates University
Email: a.jallad@uaeu.ac.ae

From: em.pone.0.8e2dc1.8abd7e87@editorialmanager.com on behalf of PLOS ONE
To: AbdulHalim Jallad
Subject: PONE-D-23-17624R3: Final Decision Being Processed - [EMID:2df8e335c1b3cb40]
Date: Thursday, September 19, 2024 10:35:51 AM

You are being carbon copied ("cc:'d") on an e-mail "To" "Firuz Kamalov" firuz@cud.ac.ae
CC: "Amril Nazir" nurmanmus@gmail.com, "Mohammad Shorfuzzaman" m.shorf@tu.edu.sa, "Muhammad Lujaini Lotfi" lujaini@analyticray.com, "Sufian Badawi" s_badawi@asu.edu.jo, "Maen Takruri" maen.takruri@aurak.ac.ae, "Abdul-Halim-M. Jallad" a.jallad@uaeu.ac.ae

Forecasting COVID-19 New Cases Using NBEATS Deep Learning and Mobility Data
PONE-D-23-17624R3

Dear Dr. Kamalov,

We're pleased to inform you that your manuscript has been judged scientifically suitable for publication and will be formally accepted for publication once it meets all outstanding technical requirements.

Within one week, you'll receive an e-mail detailing the required amendments. When these have been addressed, you'll receive a formal acceptance letter and your manuscript will be scheduled for publication.

An invoice will be generated when your article is formally accepted. Please note, if your institution has a publishing partnership with PLOS and your article meets the relevant criteria, all or part of your publication costs will be covered. Please make sure your user information is up-to-date by logging into Editorial Manager at Editorial Manager® and clicking the 'Update My Information' link at the top of the page. If you have any questions relating to publication charges, please contact our Author Billing department directly at authorbilling@plos.org.

If your institution or institutions have a press office, please notify them about your upcoming paper to help maximize its impact. If they'll be preparing press materials, please inform our press team as soon as possible -- no later than 48 hours after receiving the formal acceptance. Your manuscript will remain under strict press embargo until 2 pm Eastern Time on the date of publication. For more information, please contact onepress@plos.org.

Kind regards,

Zeyar Aung
Academic Editor
PLOS ONE

Additional Editor Comments (optional):

Three reviewers have agreed to accept the paper now. So, I follow their recommendations.

Thank you for your contribution to PLOS ONE.

Reviewers' comments:

Reviewer's Responses to Questions

Comments to the Author

1. If the authors have adequately addressed your comments raised in a previous round of review and you feel that this manuscript is now acceptable for publication, you may indicate that here to bypass the “Comments to the Author” section, enter your conflict of interest statement in the “Confidential to Editor” section, and submit your "Accept" recommendation.

Reviewer #3: All comments have been addressed

---

2. Is the manuscript technically sound, and do the data support the conclusions?

The manuscript must describe a technically sound piece of scientific research with data that supports the conclusions. Experiments must have been conducted rigorously, with appropriate controls, replication, and sample sizes. The conclusions must be drawn appropriately based on the data presented.

Reviewer #3: Yes

---

3. Has the statistical analysis been performed appropriately and rigorously?

Reviewer #3: Yes

---

4. Have the authors made all data underlying the findings in their manuscript fully available?

The PLOS Data policy requires authors to make all data underlying the findings described in their manuscript fully available without restriction, with rare exception (please refer to the Data Availability Statement in the manuscript PDF file). The data should be provided as part of the manuscript or its supporting information, or deposited to a public repository. For example, in addition to summary statistics, the data points behind means, medians and variance measures should be available. If there are restrictions on publicly sharing data—e.g. participant privacy or use of data from a third party—those must be specified.

Reviewer #3: Yes

---

5. Is the manuscript presented in an intelligible fashion and written in standard English?

PLOS ONE does not copyedit accepted manuscripts, so the language in submitted articles must be clear, correct, and unambiguous. Any typographical or grammatical errors should be corrected at revision, so please note any specific errors here.

Reviewer #3: Yes

---

6. Review Comments to the Author

Please use the space provided to explain your answers to the questions above. You may also include additional comments for the author, including concerns about dual publication, research ethics, or publication ethics. (Please upload your review as an attachment if it exceeds 20,000 characters)

Reviewer #3: (No Response)

---

7. PLOS authors have the option to publish the peer review history of their article (what does this mean?). If published, this will include your full peer review and any attached files.

If you choose "no", your identity will remain anonymous but your review may still be made public.

Do you want your identity to be public for this peer review? For information about this choice, including consent withdrawal, please see our Privacy Policy.

Reviewer #3: No

In compliance with data protection regulations, you may request that we remove your personal registration details at any time. (Remove my information/details). Please contact the publication office if you have any questions.
