# Supplementary material for: Forecasting COVID-19 new cases using NBEATS deep learning and mobility data
Source: PLoS One. 2026 Jun 29;21(6):e0350264. doi: 10.1371/journal.pone.0350264 (PMC13313360; doi:10.1371/journal.pone.0350264)
Supplement: Attachment — Submitted filename: Response_to_Reviewers_PLOSONE_COVID.pdf [file pone.0350264.s002.pdf]

Response to Reviewers' Comments

Manuscript: **Forecasting COVID-19 New Cases Using N-BEATS Deep Learning and Mobility Data**

Dear Editor and Reviewers,

We sincerely thank the Editor and all Reviewers for their careful evaluation of our manuscript and for the constructive, insightful comments. We have revised the manuscript to improve clarity, methodological transparency, and the interpretability of the reported results. Below, we provide a point-by-point response to each comment. Reviewer comments are reproduced in italics, followed by our responses describing the revisions and clarifications made in the manuscript.

Reviewer #1

Reviewer #1 – Comment 1

Clearly separate and define contrast and ablation studies within Section 4 to highlight the contribution of mobility covariates and individual model components.

Response:

We thank the reviewer for this suggestion. In Section 4 (Results and Discussion), we have clearly structured the analysis to separate the performance of the N-BEATS model with mobility covariates versus without covariates (Tables 1–4). This comparison serves as our primary ablation study, isolating the specific contribution of the mobility data to the model's forecasting accuracy. The results are analyzed in subsections 4.5.1 through 4.5.4, and the direct comparison with the LSTM-Markov baseline is detailed in subsections 4.5.5 and 4.5.6.

Reviewer #1 – Comment 2

Address unrealistic values (e.g., extremely high MAPE figures in Table 1) and ensure all reported results are verified and interpretable.

Response:

We added a focused clarification on the interpretation of MAPE in epidemiological time series. In particular, very large MAPE values can occur when the actual daily case counts approach small values, because MAPE divides by the ground-truth value at each time step.

To ensure interpretability, we now (i) explicitly explain this known behavior of MAPE, and (ii) emphasize RMSE alongside MAPE for model comparison, since RMSE is less sensitive to near-zero denominators.

Inserted clarification (Section 4.5.2 / metric interpretation):

MAPE normalizes the absolute error by the ground-truth value. When daily case counts are very small (e.g., early outbreak periods or brief low-count intervals), the denominator becomes small and the percentage error can become disproportionately large. Accordingly, MAPE is interpreted primarily for within-dataset model comparison, while RMSE is emphasized as a more stable indicator of absolute predictive accuracy in low-count regimes.

Reviewer #1 – Comment 3

Restructure Section 4 into clearly labeled subsections (e.g., Data Sources, Preprocessing, Evaluation Metrics, Experimental Results) for better readability and logical progression.

Response:

Section 4 has been reorganized into clearly labeled subsections to improve readability and logical progression. The revised structure separates (i) data sources, (ii) preprocessing, (iii) evaluation metrics, (iv) Interpretability of N-BEATS Outputs and (v) experimental results/discussion.

Reviewer #1 – Comment 4

Replace non-standard terms like "2-Shot dataset" with academically accepted alternatives such as "dual-phase evaluation" or "segmented testing."

Response:

We appreciate this feedback regarding terminology. In **Section 4.2**, we have clarified the definition of these datasets. We utilized the term "Shot" to align with the specific training/testing split methodology used in our experimental setup, where the model is tested on specific segments. The text in **Section 4.2** to ensure the distinction between the "1-Shot" (single split) and "2-Shot" (split into two distinct time series phases, Series X and Series Y) is clearly described as a data structuring strategy for validation.

Reviewer #1 – Comment 5

Provide a brief but clear rationale for selecting the USA, UK, Brazil, and Russia as study cases, considering data availability, severity, or comparability to prior work.

Response:

We added a brief rationale for selecting the USA, UK, Brazil, and Russia. The four countries were chosen because they (i) were among the most severely affected during the studied period, (ii) provide sufficient publicly available case and mobility data for consistent analysis, and (iii) offer diversity in outbreak timing and reporting patterns, supporting a more robust evaluation across different national contexts.

Where addressed in the manuscript:

Section 4.1 (Data Source) – country selection rationale paragraph:

we have selected four countries initially chosen for evaluation by [11]. These countries are the United States (USA) [48], Britain (UK) [49], Brazil [50], and Russia [51]. We chose the same countries as it would allow us to provide direct comparisons to the results from [11]. Like other Covid-19 research, these countries are the major selection countries because the affected number of people during the pandemic is high, with the most confirmed daily new cases worldwide [9].

Reviewer #1 – Comment 6

Elaborate on how the Google, Apple, and OWID datasets were filtered, merged, and aligned across countries and timeframes.

Response:

In section 4.2, we describe how features from Apple (driving, walking, transit) and Google (retail, pharmacy, parks, etc.) were filtered by country, grouped by date, and merged with the daily new cases from the OWID dataset to create a unified time-series input for the N-BEATS model.

Reviewer #1 – Comment 7

Quantify how N-BEATS improves upon the LSTM-Markov baseline across different datasets and countries, and emphasize the statistical significance of those gains.

Response:

We have included specific quantifications of the improvement in Sections 4.5.5 and 4.5.6. For example, we highlight that in the USA dataset, the RMSE of the LSTM-Markov model differs from the N-BEATS model by approximately 194%–198%. Similar substantial reductions in error rates are reported for the UK, Brazil, and Russia. The magnitude of these reductions (often reducing error by half or more) underscores the significance of the performance gain.

Where addressed in the manuscript: Section 4 (Results and Discussion) – summary paragraph comparing N-BEATS vs LSTM-Markov and with- vs without-covariates.

Reviewer #1 – Comment 8

Ensure all figures and tables are clearly labeled, interpretable, and accompanied by sufficient narrative explanation in the text.

Response:

We have ensured that all Tables (1 through 8) and Figures (1 through 5) are clearly captioned. The text in Section 4.4 explicitly references these tables to guide the reader through the comparative results for each country.

Reviewer #1 – Comment 9

Edit for grammar, sentence clarity, article use, and consistency in scientific tone throughout the manuscript.

We have conducted a thorough proofreading of the manuscript to enhance clarity and flow. We have corrected phrasing to ensure a formal academic tone (e.g., refining the Abstract and Introduction) and corrected grammatical inconsistencies.

Reviewer #1 – Comment 10

Acknowledge any dataset biases, generalizability concerns, or model limitations, and suggest how future research can extend or improve upon this work.

Response:

We have added a paragraph in **Section 1 (Introduction)**. In this paragraph, we explicitly acknowledge:

1. The focus on the early stages of the pandemic due to mobility data availability.
2. The exclusion of vaccination and population immunity data, which are critical in later stages.
3. The challenges of generalizing findings to phases where the relationship between mobility and transmission was altered by mass vaccination. We also suggest future research directions to address these gaps.

Reviewer #2

Reviewer #2 – Comment 1

Although the authors describe the experimental process using the N-BEATS model, the description of key experimental settings and data preprocessing steps is insufficient. The authors are advised to provide detailed information on data preprocessing to ensure the reproducibility of the study.

Response:

We have significantly expanded the description of our experimental setup to ensure reproducibility. In Section 4.5 (Experimental Results), we explicitly state that the N-BEATS model was trained under a fixed set of hyperparameters across all experiments to ensure consistency. We have added a specific table (Table 9) and narrative text detailing that a 30-day input window was used to predict a 14-day horizon, with 4 blocks per stack and 4 layers

per block (width 512). We also specified the use of the Adam optimizer with a learning rate of 0.001 and a dropout rate of 0.1 for regularization. Furthermore, we clarified that experiments were repeated five times with different random seeds to account for stochastic variability.

Reviewer #2 – Comment 2

The paper mentions the use of certain parameters (such as number of layers, input chunk length, and dropout rate) to balance and stabilize the performance of the N-BEATS model, but it does not elaborate on how these parameters were selected and tuned. The authors are advised to provide a detailed description of the basis for parameter selection and the tuning process, including whether cross-validation or other methods were used to determine the optimal parameter combination.

Response:

We have clarified our parameter selection strategy in **Section 3 (Methods)**. As detailed in the revised manuscript, we employed a targeted trial-and-error approach rather than an exhaustive grid search or Bayesian optimization, which would have been computationally prohibitive given the multiple countries and datasets involved. We systematically evaluated variations in the "input chunk length" parameter as it had the most significant influence on capturing temporal dynamics, selecting values that minimized RMSE and MAPE. For architectural parameters (e.g., number of stacks, blocks, and expansion coefficient dimensions), we adopted the default configuration provided by the Darts N-BEATS implementation to ensure reproducibility and reduce the risk of overfitting to the limited data.

Reviewer #2 – Comment 3

While the paper acknowledges some limitations of the study, such as the time range of the data and the lack of consideration of key factors like vaccination and herd immunity, the discussion on the specific impact of these limitations on the model's predictive accuracy is not in-depth enough.

Response:

We explicitly note that the OWID dataset contains unspecified or missing vaccination data for most countries, which necessitated its exclusion to avoid introducing bias. We acknowledge that while vaccines were administered, rapid viral mutation kept transmission a global concern. We also highlight that mobility data from Apple and Google may not represent the entire population (reflecting only smartphone users), and that reporting strategy changes (retrospective adjustments) introduce uncertainty. These factors are now contextually integrated into our analysis of the model's performance.

Reviewer #2 – Comment 4

The authors do not provide adequate explanations for the volatility and outliers in the prediction results of the N-BEATS model.

Response:

We have added a specific explanation for forecast volatility in the first paragraph of Section 4 (Results and Discussion). We explain that the emergence of COVID-19 provided limited historical references for modeling. Because N-BEATS forecasts rely heavily on patterns within the lookback window, irregularities in reported data—such as delays in reporting or sudden policy shifts—manifested as outliers. Furthermore, the model's purely data-driven design treats sharp fluctuations as signals rather than noise, and the doubly-residual stacking architecture can magnify small deviations. We clarify that many outliers coincided with the onset of major waves, suggesting they reflect real-world regime shifts rather than pure model error.

Reviewer #2 – Comment 5

Although the N-BEATS model has a certain degree of interpretability, the authors have not thoroughly explored how to use the model's output to explain the dynamic changes in the spread of COVID-19.

Response:

We have added a new subsection, 4.4 Interpretability of N-BEATS Outputs, to address this. We discuss how the model’s trend components aligned with major pandemic waves (long-term growth/decline) while seasonal components captured shorter cycles like weekly reporting effects. We provide specific examples, such as how the relatively higher RMSE in the USA during early reopening reflected abrupt changes in workplace and transit mobility, and how the stabilization following strict lockdowns in the UK was captured by the model's trend component.

Reviewer #3

Reviewer #3 – Comment 1

Lacks the Statistical Validation: While RMSE and MAPE are reported, no confidence intervals, statistical tests, or significance levels are presented.

Response:

Thank you for the important suggestion. Because COVID-19 daily case errors are temporally dependent and influenced by reporting artifacts, standard paired significance tests can be misleading without dependence-aware corrections. We therefore report RMSE/MAPE and emphasize consistency across multiple datasets/horizons, while noting formal dependence-aware statistical testing as future work.

Reviewer #3 – Comment 2

Has no Hyper-parameter Optimization Description. More detail is needed regarding how these were chosen.

Response:

Thank you for the comment. We have inserted a detailed explanation of our hyperparameter optimization in **Section 3 (Methods)**. We utilized a targeted trial-and-error approach to determine the optimal input chunk length and time-steps, prioritizing the minimization of training loss, RMSE, and MAPE. For the remaining network parameters (stacks, layers, widths), we strictly adhered to the default Darts N-BEATS configuration. This decision was made to balance computational feasibility with the need to prevent overfitting and ensure that our results are reproducible by other researchers using standard implementations.

Reviewer #3 – Comment 3

Missing Baseline Comparisons: While the focus is on LSTM-Markov vs. N-BEATS, including more traditional time series models like ARIMA as additional baselines would offer a more comprehensive benchmarking.

Response:

We have expanded the **Related Work (Section 2)** to explicitly justify our decision to exclude ARIMA as a primary baseline. We cite literature (Ma et al., Chi et al.) demonstrating that ARIMA's auto-regressive framework, which relies on linear combinations, performs poorly in long-term COVID-19 forecasting. It is unsuitable for modeling the abrupt fluctuations, structural changes, and non-stationary behavior inherent in pandemic data. Therefore, we maintained LSTM-Markov as the primary baseline because it is a more sophisticated architecture capable of better representing the complex, non-linear dynamics of the datasets used in this study.

Reviewer #3 – Comment 4

MAPE Values are Suspiciously High: The MAPE values for the UK in Table 1 are implausibly large and suggest a computational or data scaling error. This needs urgent correction.

Response:

We have reviewed the data and found that the high MAPE values in specific segments (particularly for the UK) are mathematically driven by periods where the denominator (actual cases) was near zero or extremely low during early pandemic phases or specific data splits. While these values are mathematically correct within the context of the calculation, they can be misleading. To address this, we have placed greater emphasis on the RMSE results in our discussion (Tables 2, 4, 5, and 6), as RMSE provides a more robust measure of error that is less sensitive to these near-zero anomalies.

Reviewer #3 – Comment 5

Limited Discussion of Limitations: The paper should discuss limitations, such as Potential bias in mobility data, Data quality inconsistencies between countries, and Changes in pandemic reporting strategies over time.

Response:

We have incorporated a robust discussion of these limitations in the Introduction (i.e. section 1). We explicitly state that: 1) Vaccination data was excluded due to being missing/unspecified in the OWID dataset; 2) Google/Apple mobility data reflects only smartphone users, introducing demographic bias; 3) Variations in testing capacity and case definitions across countries affect data comparability; and 4) Retrospective adjustments in reporting strategies introduce uncertainty.

Reviewer #3 – Comment 6

No Real-Time Deployment Implication: It would be useful to comment on whether this approach can be implemented in real-time.

Response:

We have updated the **Conclusion (Section 5)** to address real-time deployment. We emphasize that the deployment of mobility dataset analysis is crucial for evaluating government interventions. We note that real-time forecasting depends strongly on transparent government policies; timely communication of measures (e.g., lockdowns, reopening) allows the forecasting model to recalibrate appropriately, whereas inconsistent announcements may distort mobility patterns and reduce predictive stability.

Reviewer #3 – Comment 7

Stylistic Errors: The manuscript includes informal and sometimes unpolished language.

Response:

We have conducted a comprehensive review of the manuscript to improve the academic tone. We have refined informal phrasing (e.g., in the **Related Work** and **Introduction** sections) to ensure the language is precise, formal, and scientifically accurate.

Reviewer #3 – Comment 8

Overlong and Redundant Literature Review: The Related Work section is overly verbose and includes many tangential studies (on lifestyle changes, tourism, etc.) that don't contribute directly to forecasting models. This could be more focused.

Response:

We appreciate the reviewer's perspective on the breadth of the literature review. However, we have chosen to retain these sections as we believe they are essential for establishing the broader socio-economic context that justifies our methodological approach.

The pandemic's impact on lifestyle, tourism, and social behavior is fundamentally linked to changes in human movement patterns. By documenting these "tangential" effects, we provide the necessary scientific rationale for selecting mobility as our primary covariate. These studies illustrate that the shifts in mobility were not arbitrary but were driven by profound changes in how society functioned during the pandemic. This context is crucial for readers to understand why a deep learning model like N-BEATS—which relies on capturing complex, non-linear relationships—is required to model the interplay between human behaviour and viral transmission effectively. We believe this comprehensive overview strengthens the paper by bridging the gap between social phenomena and data-driven forecasting.
