# Supplementary material for: Forecasting COVID-19 new cases using NBEATS deep learning and mobility data
Source: PLoS One. 2026 Jun 29;21(6):e0350264. doi: 10.1371/journal.pone.0350264 (PMC13313360; doi:10.1371/journal.pone.0350264)
Supplement: Attachment — Submitted filename: Response_to_Reviewers_PLOSONE_COVID_R2_final.pdf [file pone.0350264.s003.pdf]

Response to Reviewers' Comments

Manuscript: Forecasting COVID-19 New Cases Using N-BEATS Deep Learning and Mobility Data

Dear Editor and Reviewers,

We sincerely thank the Editor and the Reviewer for the careful evaluation of our manuscript and for the constructive comments. In this revised response, we address the additional comments raised by Reviewer #2 in the latest review round. Reviewer comments are reproduced in italics, followed by our responses describing the revisions and clarifications made in the manuscript.

Reviewer #2

Reviewer #2 – Comment 1

The datasets used by the authors mainly focus on the early stages of the pandemic and lack data on key factors such as vaccination and herd immunity. This may limit the applicability and predictive accuracy of the model in the later stages of the pandemic. It is recommended that the authors provide a detailed discussion in the discussion section on the specific impact of these data limitations on the model's predictive performance and explore how to overcome these limitations in future research.

Response: We thank the reviewer for this important observation. In the revised manuscript, we have added a dedicated discussion paragraph clarifying that the datasets used in this study mainly represent the earlier phases of the COVID-19 pandemic, before vaccination coverage, booster uptake, herd immunity, and variant-specific effects became increasingly important drivers of case dynamics. We now explain that, as a consequence, the relationship learned by the model between mobility patterns and daily new cases is likely to be more reliable for early-wave dynamics than for later pandemic stages. We further note that, in later phases, case trends were increasingly shaped by vaccination, waning immunity, emerging variants, testing practices, and changing public-health interventions, which may reduce predictive accuracy and limit temporal generalizability. Finally, we added future-work directions indicating that vaccination-related, immunity-related, variant-related, testing, and policy-intervention variables should be incorporated in subsequent studies to improve robustness across evolving epidemic phases.

Reviewer #2 – Comment 2

Although the authors reported indicators such as RMSE and MAPE, they did not provide statistical validation such as confidence intervals, statistical tests, or significance levels. It is recommended that the authors conduct dependence-corrected statistical tests to more comprehensively evaluate model performance.

Response: We thank the reviewer for this comment. We agree that the previous version of the manuscript did not provide formal statistical validation such as confidence intervals, significance levels, or dependence-corrected forecast-comparison tests. In the revised manuscript, we have clarified that the reported RMSE and MAPE values are based on experiments repeated five times with different random seeds, and that the reported results correspond to averages across runs. Accordingly, we have revised the manuscript to avoid overstating statistical significance and now describe the results as showing consistent empirical performance improvements rather than formally confirmed statistical significance. In particular, we revised the Abstract and Conclusion to replace significance-based wording with more precise comparative language. We agree that dependence-corrected statistical testing would further strengthen the evaluation, and we identify this as an important direction for future work.

Reviewer #2 – Comment 3

The authors used a trial-and-error approach in selecting model parameters but did not elaborate on this process. It is recommended that the authors provide a more detailed description of hyperparameter optimization, including how to determine the optimal parameter combination and whether cross-validation or other methods were used.

Response: We appreciate the reviewer's helpful suggestion. We agree that the earlier description of hyperparameter selection was not sufficiently detailed. In the revised manuscript, we clarify that parameter selection was performed using a targeted trial-and-error procedure rather than exhaustive search, and that input chunk length and forecast time-step settings were examined because they had the strongest influence on predictive performance. Candidate settings were assessed using RMSE, MAPE, and training loss, and the final configuration was selected based on overall predictive performance. These additions improve the transparency of the model-selection procedure.
