# Supplementary material for: Forecasting COVID-19 new cases using NBEATS deep learning and mobility data
Source: PLoS One. 2026 Jun 29;21(6):e0350264. doi: 10.1371/journal.pone.0350264 (PMC13313360; doi:10.1371/journal.pone.0350264)
Supplement: Peer review history

# Author comment: Author response to Decision Letter 0

**DOI:** 10.1371/journal.pone.0350264.r001

12 Jun 2025

Attachment Submitted filename: Binder1.pdf

# Peer review: Decision Letter 0

**DOI:** 10.1371/journal.pone.0350264.r002

**Authors:** Muhammad Ahsan

25 Jul 2025

PONE-D-25-27036

Forecasting COVID-19 New Cases Using NBEATS Deep Learning and Mobility Data

PLOS ONE

Dear Dr. Jallad,

Thank you for submitting your manuscript to PLOS ONE. After careful consideration, we feel that it has merit but does not fully meet PLOS ONE’s publication criteria as it currently stands. Therefore, we invite you to submit a revised version of the manuscript that addresses the points raised during the review process.

Please submit your revised manuscript by Sep 08 2025 11:59PM. If you will need more time than this to complete your revisions, please reply to this message or contact the journal office at plosone@plos.org. When you're ready to submit your revision, log on to https://www.editorialmanager.com/pone/ and select the 'Submissions Needing Revision' folder to locate your manuscript file.

Please include the following items when submitting your revised manuscript:

- A rebuttal letter that responds to each point raised by the academic editor and reviewer(s). You should upload this letter as a separate file labeled 'Response to Reviewers'.
- A marked-up copy of your manuscript that highlights changes made to the original version. You should upload this as a separate file labeled 'Revised Manuscript with Track Changes'.
- An unmarked version of your revised paper without tracked changes. You should upload this as a separate file labeled 'Manuscript'.

If you would like to make changes to your financial disclosure, please include your updated statement in your cover letter. Guidelines for resubmitting your figure files are available below the reviewer comments at the end of this letter.

If applicable, we recommend that you deposit your laboratory protocols in protocols.io to enhance the reproducibility of your results. Protocols.io assigns your protocol its own identifier (DOI) so that it can be cited independently in the future. For instructions see: https://journals.plos.org/plosone/s/submission-guidelines#loc-laboratory-protocols. Additionally, PLOS ONE offers an option for publishing peer-reviewed Lab Protocol articles, which describe protocols hosted on protocols.io. Read more information on sharing protocols at https://plos.org/protocols?utm_medium=editorial-email&utm_source=authorletters&utm_campaign=protocols.

We look forward to receiving your revised manuscript.

Kind regards,

Muhammad Ahsan, Ph.D.

Academic Editor

PLOS ONE

Journal Requirements:

When submitting your revision, we need you to address these additional requirements.

1. Please ensure that your manuscript meets PLOS ONE's style requirements, including those for file naming. The PLOS ONE style templates can be found at

https://journals.plos.org/plosone/s/file?id=wjVg/PLOSOne_formatting_sample_main_body.pdf and

https://journals.plos.org/plosone/s/file?id=ba62/PLOSOne_formatting_sample_title_authors_affiliations.pdf.

2. Please note that PLOS One has specific guidelines on code sharing for submissions in which author-generated code underpins the findings in the manuscript. In these cases, we expect all author-generated code to be made available without restrictions upon publication of the work. Please review our guidelines at https://journals.plos.org/plosone/s/materials-and-software-sharing#loc-sharing-code and ensure that your code is shared in a way that follows best practice and facilitates reproducibility and reuse.

3.  We note that the grant information you provided in the ‘Funding Information’ and ‘Financial Disclosure’ sections do not match.

When you resubmit, please ensure that you provide the correct grant numbers for the awards you received for your study in the ‘Funding Information’ section.

4. If the reviewer comments include a recommendation to cite specific previously published works, please review and evaluate these publications to determine whether they are relevant and should be cited. There is no requirement to cite these works unless the editor has indicated otherwise.

[Note: HTML markup is below. Please do not edit.]

Reviewers' comments:

Reviewer's Responses to Questions

**Comments to the Author**

1. Is the manuscript technically sound, and do the data support the conclusions?

The manuscript must describe a technically sound piece of scientific research with data that supports the conclusions. Experiments must have been conducted rigorously, with appropriate controls, replication, and sample sizes. The conclusions must be drawn appropriately based on the data presented.

Reviewer #1: Yes

Reviewer #2: Partly

Reviewer #3: Yes

**********

2. Has the statistical analysis been performed appropriately and rigorously?

Reviewer #1: Yes

Reviewer #2: Yes

Reviewer #3: Yes

**********

3. Have the authors made all data underlying the findings in their manuscript fully available?

The PLOS Data policy requires authors to make all data underlying the findings described in their manuscript fully available without restriction, with rare exception (please refer to the Data Availability Statement in the manuscript PDF file). The data should be provided as part of the manuscript or its supporting information, or deposited to a public repository. For example, in addition to summary statistics, the data points behind means, medians and variance measures should be available. If there are restrictions on publicly sharing data—e.g. participant privacy or use of data from a third party—those must be specified.

Reviewer #1: Yes

Reviewer #2: Yes

Reviewer #3: Yes

**********

4. Is the manuscript presented in an intelligible fashion and written in standard English?

PLOS ONE does not copyedit accepted manuscripts, so the language in submitted articles must be clear, correct, and unambiguous. Any typographical or grammatical errors should be corrected at revision, so please note any specific errors here.

Reviewer #1: Yes

Reviewer #2: Yes

Reviewer #3: Yes

**********

5. Review Comments to the Author

Please use the space provided to explain your answers to the questions above. You may also include additional comments for the author, including concerns about dual publication, research ethics, or publication ethics. (Please upload your review as an attachment if it exceeds 20,000 characters)

Reviewer #1: This study proposes a deep learning-based approach using the N-BEATS architecture to forecast daily new COVID-19 cases by integrating mobility data from Google and Apple. The model is tested on data from four heavily impacted countries — the USA, UK, Brazil, and Russia — and is evaluated against a benchmark LSTM-Markov model. The results show that N-BEATS, especially when combined with mobility covariates, significantly outperforms the baseline in terms of RMSE and MAPE. However, the study faces several limitations that could be addressed in future revisions: the absence of a clearly structured empirical analysis (e.g., ablation and contrast studies), lack of justification for country selection, and numerical anomalies in the reported results. Addressing these limitations would strengthen the model's generalizability and the paper’s overall scientific contribution. The research nonetheless highlights the critical role of mobility trends in understanding pandemic dynamics and offers valuable insights for policymakers. Authors should should address the following key points to make the paper suitable for publication:

1. Clearly separate and define contrast and ablation studies within Section 4 to highlight the contribution of mobility covariates and individual model components.

2. Address unrealistic values (e.g., extremely high MAPE figures in Table 1) and ensure all reported results are verified and interpretable.

3. Restructure Section 4 into clearly labeled subsections (e.g., Data Sources, Preprocessing, Evaluation Metrics, Experimental Results) for better readability and logical progression.

4. Replace non-standard terms like "2-Shot dataset" with academically accepted alternatives such as “dual-phase evaluation” or “segmented testing.”

5. Provide a brief but clear rationale for selecting the USA, UK, Brazil, and Russia as study cases, considering data availability, severity, or comparability to prior work.

6. Elaborate on how the Google, Apple, and OWID datasets were filtered, merged, and aligned across countries and timeframes.

7. Quantify how N-BEATS improves upon the LSTM-Markov baseline across different datasets and countries, and emphasize the statistical significance of those gains.

8. Ensure all figures and tables are clearly labeled, interpretable, and accompanied by sufficient narrative explanation in the text.

9. Edit for grammar, sentence clarity, article use, and consistency in scientific tone throughout the manuscript.

10. Acknowledge any dataset biases, generalizability concerns, or model limitations, and suggest how future research can extend or improve upon this work.

Reviewer #2: Title: Forecasting COVID-19 New Cases Using NBEATS Deep Learning and Mobility Data

Manuscript Number: PONE-D-25-27036

This paper proposes a COVID-19 new case forecasting method based on the N-BEATS deep learning architecture and incorporates mobility data from Google and Apple to enhance the model's accuracy and interpretability. The study selects four countries severely affected by the pandemic— the United States, Russia, the United Kingdom, and Brazil — for experimentation and evaluates the model using three distinct datasets from Google, Apple, and OWID. The results demonstrate that the N-BEATS model outperforms the existing LSTM-Markov model across all datasets, significantly reducing the Root Mean Squared Error (RMSE) and Mean Absolute Percentage Error (MAPE). Moreover, the N-BEATS model with mobility data as covariates outperforms its counterpart without covariates, highlighting the importance of mobility data in predicting the spread of COVID-19. The main contributions of the paper lie in verifying the effectiveness of the N-BEATS architecture in forecasting COVID-19 cases and demonstrating the critical role of mobility data in understanding the dynamics of the pandemic. The study offers valuable insights for policymakers and health officials to better manage future pandemics.

However, despite the paper's innovation and rigor in methodology and experimental design, there are areas for further improvement:

1. Although the authors describe the experimental process using the N-BEATS model, the description of key experimental settings and data preprocessing steps is insufficient. The authors are advised to provide detailed information on data preprocessing to ensure the reproducibility of the study.

2. The paper mentions the use of certain parameters (such as number of layers, input chunk length, and dropout rate) to balance and stabilize the performance of the N-BEATS model, but it does not elaborate on how these parameters were selected and tuned. The authors are advised to provide a detailed description of the basis for parameter selection and the tuning process, including whether cross-validation or other methods were used to determine the optimal parameter combination.

3. While the paper acknowledges some limitations of the study, such as the time range of the data and the lack of consideration of key factors like vaccination and herd immunity, the discussion on the specific impact of these limitations on the model's predictive accuracy is not in-depth enough.

4. The authors do not provide adequate explanations for the volatility and outliers in the prediction results of the N-BEATS model.

5. Although the N-BEATS model has a certain degree of interpretability, the authors have not thoroughly explored how to use the model's output to explain the dynamic changes in the spread of COVID-19.

Reviewer #3: The manuscript introduces a deep learning-based approach using the N-BEATS architecture to forecast COVID-19 daily new cases by integrating mobility data. The study compares N-BEATS performance to LSTM-Markov across datasets and countries. It demonstrates promising results, particularly in leveraging mobility data to enhance forecast accuracy. However the research

1. Lacks the Statistical Validation: While RMSE and MAPE are reported, no confidence intervals, statistical tests, or significance levels are presented. A paired t-test or Wilcoxon signed-rank test would help confirm whether the improvements by N-BEATS over LSTM-Markov are statistically significant.

2. Has no Hyper-parameter Optimization Description: The paper mentions using default parameters for the N-BEATS model. More detail is needed regarding how these were chosen and whether hyper-parameter tuning via grid search or Bayesian optimization was explored.

3. Missing Baseline Comparisons: While the focus is on LSTM-Markov vs. N-BEATS, including more traditional time series models like ARIMA as additional baselines would offer a more comprehensive benchmarking.

4. MAPE Values are Suspiciously High: The MAPE values for the UK in Table 1 are implausibly large and suggest a computational or data scaling error. This needs urgent correction.

5. Limited Discussion of Limitations: The paper should discuss limitations, such as Potential bias in mobility data, Data quality inconsistencies between countries, and Changes in pandemic reporting strategies over time.

6. No Real-Time Deployment Implication: It would be useful to comment on whether this approach can be implemented in real-time and how it would adapt to new variants or changing public health policies.

7. Stylistic Errors: The manuscript includes informal and sometimes unpolished language like “debilitated by the closure…” or “vast of population lost their lives”, which should be revised for academic tone and clarity.

8. Overlong and Redundant Literature Review: The Related Work section is overly verbose and includes many tangential studies (on lifestyle changes, tourism, etc.) that don’t contribute directly to forecasting models. This could be more focused.

Specific Recommendations:

1. Revise Table 1 to remove implausible MAPE values, validate correctness.

2. Add a statistical test comparing model performances.

3. Discuss model limitations, especially related to generalizability and real-time forecasting.

4. Provide details on how data preprocessing handled missing values, inconsistencies, and scaling.

5. Improve writing clarity and reduce redundancy in the Related Work section.

6. Shorten or summarize repetitive parts of the results and avoid excessive figures with very similar trends.

7. Add code or pseudo-code for reproducibility, if possible.

**********

6. PLOS authors have the option to publish the peer review history of their article (what does this mean?). If published, this will include your full peer review and any attached files.

If you choose “no”, your identity will remain anonymous but your review may still be made public.

**Do you want your identity to be public for this peer review?**  For information about this choice, including consent withdrawal, please see our Privacy Policy.

Reviewer #1: **Yes:** sushree Subhaprada pradhan

Reviewer #2: No

Reviewer #3: No

**********

[NOTE: If reviewer comments were submitted as an attachment file, they will be attached to this email and accessible via the submission site. Please log into your account, locate the manuscript record, and check for the action link "View Attachments". If this link does not appear, there are no attachment files.]

While revising your submission, please upload your figure files to the Preflight Analysis and Conversion Engine (PACE) digital diagnostic tool, https://pacev2.apexcovantage.com/. PACE helps ensure that figures meet PLOS requirements. To use PACE, you must first register as a user. Registration is free. Then, login and navigate to the UPLOAD tab, where you will find detailed instructions on how to use the tool. If you encounter any issues or have any questions when using PACE, please email PLOS at figures@plos.org. Please note that Supporting Information files do not need this step.

# Author comment: Author response to Decision Letter 1

**DOI:** 10.1371/journal.pone.0350264.r003

11 Feb 2026

Response to Reviewers’ Comments

Manuscript: Forecasting COVID-19 New Cases Using N-BEATS Deep Learning and Mobility Data

Dear Editor and Reviewers,

We sincerely thank the Editor and all Reviewers for their careful evaluation of our manuscript and for the constructive, insightful comments. We have revised the manuscript to improve clarity, methodological transparency, and the interpretability of the reported results. Below, we provide a point‑by‑point response to each comment. Reviewer comments are reproduced in italics, followed by our responses describing the revisions and clarifications made in the manuscript.

Reviewer #1

Reviewer #1 – Comment 1

Clearly separate and define contrast and ablation studies within Section 4 to highlight the contribution of mobility covariates and individual model components.

Response:

We thank the reviewer for this suggestion. In Section 4 (Results and Discussion), we have clearly structured the analysis to separate the performance of the N-BEATS model with mobility covariates versus without covariates (Tables 1–4). This comparison serves as our primary ablation study, isolating the specific contribution of the mobility data to the model's forecasting accuracy. The results are analyzed in subsections 4.5.1 through 4.5.4, and the direct comparison with the LSTM-Markov baseline is detailed in subsections 4.5.5 and 4.5.6.

Reviewer #1 – Comment 2

Address unrealistic values (e.g., extremely high MAPE figures in Table 1) and ensure all reported results are verified and interpretable.

Response:

We added a focused clarification on the interpretation of MAPE in epidemiological time series. In particular, very large MAPE values can occur when the actual daily case counts approach small values, because MAPE divides by the ground‑truth value at each time step.

To ensure interpretability, we now (i) explicitly explain this known behavior of MAPE, and (ii) emphasize RMSE alongside MAPE for model comparison, since RMSE is less sensitive to near‑zero denominators.

Inserted clarification (Section 4.5.2 / metric interpretation):

MAPE normalizes the absolute error by the ground‑truth value. When daily case counts are very small (e.g., early outbreak periods or brief low‑count intervals), the denominator becomes small and the percentage error can become disproportionately large. Accordingly, MAPE is interpreted primarily for within‑dataset model comparison, while RMSE is emphasized as a more stable indicator of absolute predictive accuracy in low‑count regimes.

Reviewer #1 – Comment 3

Restructure Section 4 into clearly labeled subsections (e.g., Data Sources, Preprocessing, Evaluation Metrics, Experimental Results) for better readability and logical progression.

Response:

Section 4 has been reorganized into clearly labeled subsections to improve readability and logical progression. The revised structure separates (i) data sources, (ii) preprocessing, (iii) evaluation metrics, (iv) Interpretability of N-BEATS Outputs and (v) experimental results/discussion.

Reviewer #1 – Comment 4

Replace non-standard terms like "2-Shot dataset" with academically accepted alternatives such as “dual-phase evaluation” or “segmented testing.”

Response:

We appreciate this feedback regarding terminology. In Section 4.2, we have clarified the definition of these datasets. We utilized the term "Shot" to align with the specific training/testing split methodology used in our experimental setup, where the model is tested on specific segments. The text in Section 4.2 to ensure the distinction between the "1-Shot" (single split) and "2-Shot" (split into two distinct time series phases, Series X and Series Y) is clearly described as a data structuring strategy for validation.

Reviewer #1 – Comment 5

Provide a brief but clear rationale for selecting the USA, UK, Brazil, and Russia as study cases, considering data availability, severity, or comparability to prior work.

Response:

We added a brief rationale for selecting the USA, UK, Brazil, and Russia. The four countries were chosen because they (i) were among the most severely affected during the studied period, (ii) provide sufficient publicly available case and mobility data for consistent analysis, and (iii) offer diversity in outbreak timing and reporting patterns, supporting a more robust evaluation across different national contexts.

Where addressed in the manuscript:

Section 4.1 (Data Source) – country selection rationale paragraph:

we have selected four countries initially chosen for evaluation by [11]. These countries are the United States (USA) [48], Britain (UK) [49], Brazil [50], and Russia [51]. We chose the same countries as it would allow us to provide direct comparisons to the results from [11]. Like other Covid-19 research, these countries are the major selection countries because the affected number of people during the pandemic is high, with the most confirmed daily new cases worldwide [9].

Reviewer #1 – Comment 6

Elaborate on how the Google, Apple, and OWID datasets were filtered, merged, and aligned across countries and timeframes.

Response:

In section 4.2, we describe how features from Apple (driving, walking, transit) and Google (retail, pharmacy, parks, etc.) were filtered by country, grouped by date, and merged with the daily new cases from the OWID dataset to create a unified time-series input for the N-BEATS model.

Reviewer #1 – Comment 7

Quantify how N-BEATS improves upon the LSTM-Markov baseline across different datasets and countries, and emphasize the statistical significance of those gains.

Response:

We have included specific quantifications of the improvement in Sections 4.5.5 and 4.5.6. For example, we highlight that in the USA dataset, the RMSE of the LSTM-Markov model differs from the N-BEATS model by approximately 194%–198%. Similar substantial reductions in error rates are reported for the UK, Brazil, and Russia. The magnitude of these reductions (often reducing error by half or more) underscores the significance of the performance gain.

Where addressed in the manuscript: Section 4 (Results and Discussion) – summary paragraph comparing N‑BEATS vs LSTM‑Markov and with‑ vs without‑covariates.

Reviewer #1 – Comment 8

Ensure all figures and tables are clearly labeled, interpretable, and accompanied by sufficient narrative explanation in the text.

Response:

We have ensured that all Tables (1 through 8) and Figures (1 through 5) are clearly captioned. The text in Section 4.4 explicitly references these tables to guide the reader through the comparative results for each country.

Reviewer #1 – Comment 9

Edit for grammar, sentence clarity, article use, and consistency in scientific tone throughout the manuscript.

We have conducted a thorough proofreading of the manuscript to enhance clarity and flow. We have corrected phrasing to ensure a formal academic tone (e.g., refining the Abstract and Introduction) and corrected grammatical inconsistencies.

Reviewer #1 – Comment 10

Acknowledge any dataset biases, generalizability concerns, or model limitations, and suggest how future research can extend or improve upon this work.

Response:

We have added a paragraph in Section 1 (Introduction). In this paragraph, we explicitly acknowledge:

1. The focus on the early stages of the pandemic due to mobility data availability.

2. The exclusion of vaccination and population immunity data, which are critical in later stages.

3. The challenges of generalizing findings to phases where the relationship between mobility and transmission was altered by mass vaccination. We also suggest future research directions to address these gaps.

Reviewer #2

Reviewer #2 – Comment 1

Although the authors describe the experimental process using the N-BEATS model, the description of key experimental settings and data preprocessing steps is insufficient. The authors are advised to provide detailed information on data preprocessing to ensure the reproducibility of the study.

Response:

We have significantly expanded the description of our experimental setup to ensure reproducibility. In Section 4.5 (Experimental Results), we explicitly state that the N-BEATS model was trained under a fixed set of hyperparameters across all experiments to ensure consistency. We have added a specific table (Table 9) and narrative text detailing that a 30-day input window was used to predict a 14-day horizon, with 4 blocks per stack and 4 layers per block (width 512). We also specified the use of the Adam optimizer with a learning rate of 0.001 and a dropout rate of 0.1 for regularization. Furthermore, we clarified that experiments were repeated five times with different random seeds to account for stochastic variability.

Reviewer #2 – Comment 2

The paper mentions the use of certain parameters (such as number of layers, input chunk length, and dropout rate) to balance and stabilize the performance of the N-BEATS model, but it does not elaborate on how these parameters were selected and tuned. The authors are advised to provide a detailed description of the basis for parameter selection and the tuning process, including whether cross-validation or other methods were used to determine the optimal parameter combination.

Response:

We have clarified our parameter selection strategy in Section 3 (Methods). As detailed in the revised manuscript, we employed a targeted trial-and-error approach rather than an exhaustive grid search or Bayesian optimization, which would have been computationally prohibitive given the multiple countries and datasets involved. We systematically evaluated variations in the "input chunk length" parameter as it had the most significant influence on capturing temporal dynamics, selecting values that minimized RMSE and MAPE. For architectural parameters (e.g., number of stacks, blocks, and expansion coefficient dimensions), we adopted the default configuration provided by the Darts N-BEATS implementation to ensure reproducibility and reduce the risk of overfitting to the limited data.

Reviewer #2 – Comment 3

While the paper acknowledges some limitations of the study, such as the time range of the data and the lack of consideration of key factors like vaccination and herd immunity, the discussion on the specific impact of these limitations on the model's predictive accuracy is not in-depth enough.

Response:

We explicitly note that the OWID dataset contains unspecified or missing vaccination data for most countries, which necessitated its exclusion to avoid introducing bias. We acknowledge that while vaccines were administered, rapid viral mutation kept transmission a global concern. We also highlight that mobility data from Apple and Google may not represent the entire population (reflecting only smartphone users), and that reporting strategy changes (retrospective adjustments) introduce uncertainty. These factors are now contextually integrated into our analysis of the model's performance.

Reviewer #2 – Comment 4

The authors do not provide adequate explanations for the volatility and outliers in the prediction results of the N-BEATS model.

Response:

We have added a specific explanation for forecast volatility in the first paragraph of Section 4 (Results and Discussion). We explain that the emergence of COVID-19 provided limited historical references for modeling. Because N-BEATS forecasts rely heavily on patterns within the lookback window, irregularities in reported data—such as delays in reporting or sudden policy shifts—manifested as outliers. Furthermore, the model's purely data-driven design treats sharp fluctuations as signals rather than noise, and the doubly-residual stacking architecture can magnify small deviations. We clarify that many outliers coincided with the onset of major waves, suggesting they reflect real-world regime shifts rather than pure model error.

Reviewer #2 – Comment 5

Although the N-BEATS model has a certain degree of interpretability, the authors have not thoroughly explored how to use the model's output to explain the dynamic changes in the spread of COVID-19.

Response:

We have added a new subsection, 4.4 Interpretability of N-BEATS Outputs, to address this. We discuss how the model’s trend components aligned with major pandemic waves (long-term growth/decline) while seasonal components captured shorter cycles like weekly reporting effects. We provide specific examples, such as how the relatively higher RMSE in the USA during early reopening reflected abrupt changes in workplace and transit mobility, and how the stabilization following strict lockdowns in the UK was captured by the model's trend component.

Reviewer #3

Reviewer #3 – Comment 1

Lacks the Statistical Validation: While RMSE and MAPE are reported, no confidence intervals, statistical tests, or significance levels are presented.

Response:

Thank you for the important suggestion. Because COVID-19 daily case errors are temporally dependent and influenced by reporting artifacts, standard paired significance tests can be misleading without dependence-aware corrections. We therefore report RMSE/MAPE and emphasize consistency across multiple datasets/horizons, while noting formal dependence-aware statistical testing as future work.

Reviewer #3 – Comment 2

Has no Hyper-parameter Optimization Description. More detail is needed regarding how these were chosen.

Response:

Thank you for the comment. We have inserted a detailed explanation of our hyperparameter optimization in Section 3 (Methods). We utilized a targeted trial-and-error approach to determine the optimal input chunk length and time-steps, prioritizing the minimization of training loss, RMSE, and MAPE. For the remaining network parameters (stacks, layers, widths), we strictly adhered to the default Darts N-BEATS configuration. This decision was made to balance computational feasibility with the need to prevent overfitting and ensure that our results are reproducible by other researchers using standard implementations.

Reviewer #3 – Comment 3

Missing Baseline Comparisons: While the focus is on LSTM-Markov vs. N-BEATS, including more traditional time series models like ARIMA as additional baselines would offer a more comprehensive benchmarking.

Response:

We have expanded the Related Work (Section 2) to explicitly justify our decision to exclude ARIMA as a primary baseline. We cite literature (Ma et al., Chi et al.) demonstrating that ARIMA's auto-regressive framework, which relies on linear combinations, performs poorly in long-term COVID-19 forecasting. It is unsuitable for modeling the abrupt fluctuations, structural changes, and non-stationary behavior inherent in pandemic data. Therefore, we maintained LSTM-Markov as the primary baseline because it is a more sophisticated architecture capable of better representing the complex, non-linear dynamics of the datasets used in this study.

Reviewer #3 – Comment 4

MAPE Values are Suspiciously High: The MAPE values for the UK in Table 1 are implausibly large and suggest a computational or data scaling error. This needs urgent correction.

Response:

We have reviewed the data and found that the high MAPE values in specific segments (particularly for the UK) are mathematically driven by periods where the denominator (actual cases) was near zero or extremely low during early pandemic phases or specific data splits. While these values are mathematically correct within the context of the calculation, they can be misleading. To address this, we have placed greater emphasis on the RMSE results in our discussion (Tables 2, 4, 5, and 6), as RMSE provides a more robust measure of error that is less sensitive to these near-zero anomalies.

Reviewer #3 – Comment 5

Limited Discussion of Limitations: The paper should discuss limitations, such as Potential bias in mobility data, Data quality inconsistencies between countries, and Changes in pandemic reporting strategies over time.

Response:

We have incorporated a robust discussion of these limitations in the Introduction (i.e. section 1). We explicitly state that: 1) Vaccination data was excluded due to being missing/unspecified in the OWID dataset; 2) Google/Apple mobility data reflects only smartphone users, introducing demographic bias; 3) Variations in testing capacity and case definitions across countries affect data com

Attachment Submitted filename: Response_to_Reviewers_PLOSONE_COVID.pdf

# Peer review: Decision Letter 1

**DOI:** 10.1371/journal.pone.0350264.r004

**Authors:** Jie Zhang

2 Mar 2026

PONE-D-25-27036R1

Forecasting COVID-19 New Cases Using NBEATS Deep Learning and Mobility Data

PLOS One

Dear Dr. Jallad,

Thank you for submitting your manuscript to PLOS ONE. After careful consideration, we feel that it has merit but does not fully meet PLOS ONE’s publication criteria as it currently stands. Therefore, we invite you to submit a revised version of the manuscript that addresses the points raised during the review process.

Please submit your revised manuscript by Apr 16 2026 11:59PM. If you will need more time than this to complete your revisions, please reply to this message or contact the journal office at plosone@plos.org. When you're ready to submit your revision, log on to https://www.editorialmanager.com/pone/ and select the 'Submissions Needing Revision' folder to locate your manuscript file.

Please include the following items when submitting your revised manuscript:

- A letter that responds to each point raised by the academic editor and reviewer(s). You should upload this letter as a separate file labeled 'Response to Reviewers'.
- A marked-up copy of your manuscript that highlights changes made to the original version. You should upload this as a separate file labeled 'Revised Manuscript with Track Changes'.
- An unmarked version of your revised paper without tracked changes. You should upload this as a separate file labeled 'Manuscript'.

-->If you would like to make changes to your financial disclosure, please include your updated statement in your cover letter. Guidelines for resubmitting your figure files are available below the reviewer comments at the end of this letter.

If applicable, we recommend that you deposit your laboratory protocols in protocols.io to enhance the reproducibility of your results. Protocols.io assigns your protocol its own identifier (DOI) so that it can be cited independently in the future. For instructions see: https://journals.plos.org/plosone/s/submission-guidelines#loc-laboratory-protocols. Additionally, PLOS ONE offers an option for publishing peer-reviewed Lab Protocol articles, which describe protocols hosted on protocols.io. Read more information on sharing protocols at https://plos.org/protocols?utm_medium=editorial-email&utm_source=authorletters&utm_campaign=protocols.

We look forward to receiving your revised manuscript.

Kind regards,

Jie Zhang

Academic Editor

PLOS One

**Journal Requirements:**

1. If the reviewer comments include a recommendation to cite specific previously published works, please review and evaluate these publications to determine whether they are relevant and should be cited. There is no requirement to cite these works unless the editor has indicated otherwise.

2. Please review your reference list to ensure that it is complete and correct. If you have cited papers that have been retracted, please include the rationale for doing so in the manuscript text, or remove these references and replace them with relevant current references. Any changes to the reference list should be mentioned in the rebuttal letter that accompanies your revised manuscript. If you need to cite a retracted article, indicate the article’s retracted status in the References list and also include a citation and full reference for the retraction notice.

**Additional Editor Comments:**

Please further revise the paper by considering the reviewer's comments.

[Note: HTML markup is below. Please do not edit.]

Reviewers' comments:

Reviewer's Responses to Questions

**Comments to the Author**

1. If the authors have adequately addressed your comments raised in a previous round of review and you feel that this manuscript is now acceptable for publication, you may indicate that here to bypass the “Comments to the Author” section, enter your conflict of interest statement in the “Confidential to Editor” section, and submit your "Accept" recommendation.

Reviewer #1: All comments have been addressed

Reviewer #2: (No Response)

Reviewer #3: All comments have been addressed

**********

2. Is the manuscript technically sound, and do the data support the conclusions?

The manuscript must describe a technically sound piece of scientific research with data that supports the conclusions. Experiments must have been conducted rigorously, with appropriate controls, replication, and sample sizes. The conclusions must be drawn appropriately based on the data presented.

Reviewer #1: Yes

Reviewer #2: (No Response)

Reviewer #3: Yes

**********

3. Has the statistical analysis been performed appropriately and rigorously?

Reviewer #1: Yes

Reviewer #2: (No Response)

Reviewer #3: Yes

**********

4. Have the authors made all data underlying the findings in their manuscript fully available?

The PLOS Data policy requires authors to make all data underlying the findings described in their manuscript fully available without restriction, with rare exception (please refer to the Data Availability Statement in the manuscript PDF file). The data should be provided as part of the manuscript or its supporting information, or deposited to a public repository. For example, in addition to summary statistics, the data points behind means, medians and variance measures should be available. If there are restrictions on publicly sharing data—e.g. participant privacy or use of data from a third party—those must be specified.

Reviewer #1: Yes

Reviewer #2: (No Response)

Reviewer #3: Yes

**********

5. Is the manuscript presented in an intelligible fashion and written in standard English?

PLOS ONE does not copyedit accepted manuscripts, so the language in submitted articles must be clear, correct, and unambiguous. Any typographical or grammatical errors should be corrected at revision, so please note any specific errors here.

Reviewer #1: Yes

Reviewer #2: (No Response)

Reviewer #3: Yes

**********

6. Review Comments to the Author

Please use the space provided to explain your answers to the questions above. You may also include additional comments for the author, including concerns about dual publication, research ethics, or publication ethics. (Please upload your review as an attachment if it exceeds 20,000 characters)

Reviewer #1: The revised manuscript titled “Forecasting COVID-19 New Cases Using N-BEATS Deep Learning and Mobility Data” has substantially improved in response to the reviewers’ comments. The authors have carefully addressed all major methodological, structural, and interpretative concerns raised during the review process. Hence it is ready for publication.

Reviewer #2: Title: Forecasting COVID-19 New Cases Using NBEATS Deep Learning and Mobility Data

Manuscript Number: PONE-D-25-27036R1

This paper proposes a novel forecasting method for COVID-19 new cases based on the N-BEATS deep learning architecture, incorporating mobility data from Google and Apple to enhance the model's accuracy and interpretability. The study selected four countries severely affected by the pandemic— the United States, the United Kingdom, Brazil, and Russia— for experimentation and evaluated the model using three distinct COVID-19 datasets from Google, Apple, and Our World in Data (OWID). The results demonstrate that the N-BEATS model outperforms the existing LSTM-Markov model across all datasets, significantly reducing the Root Mean Squared Error (RMSE) and Mean Absolute Percentage Error (MAPE). Moreover, the N-BEATS model with mobility data as covariates outperforms its counterpart without covariates, highlighting the importance of mobility data in predicting the spread of COVID-19. The main contribution of this paper lies in verifying the effectiveness of the N-BEATS architecture in forecasting COVID-19 cases and demonstrating the critical role of mobility data in understanding the dynamics of the pandemic. The study provides valuable insights for policymakers and public health officials to better manage future pandemics. The authors have made substantial improvements in the experimental setup, data preprocessing, model parameter selection, and result interpretation in response to the previous review comments, significantly enhancing the quality and readability of the manuscript. Despite the many improvements made by the authors in response to the previous review comments, there is still room for further refinement of the paper:

1. The datasets used by the authors mainly focus on the early stages of the pandemic and lack data on key factors such as vaccination and herd immunity. This may limit the applicability and predictive accuracy of the model in the later stages of the pandemic. It is recommended that the authors provide a detailed discussion in the discussion section on the specific impact of these data limitations on the model's predictive performance and explore how to overcome these limitations in future research.

2. Although the authors reported indicators such as RMSE and MAPE, they did not provide statistical validation such as confidence intervals, statistical tests, or significance levels. It is recommended that the authors conduct dependence-corrected statistical tests to more comprehensively evaluate model performance.

3. The authors used a trial-and-error approach in selecting model parameters but did not elaborate on this process. It is recommended that the authors provide a more detailed description of hyperparameter optimization, including how to determine the optimal parameter combination and whether cross-validation or other methods were used.

Reviewer #3: The authors have substantially improved the manuscript in response to the previous review. The major methodological and presentation-related concerns have been adequately addressed. The revised version shows clearer exposition, corrected numerical results, and improved rigor in experimental validation.

**********

7. PLOS authors have the option to publish the peer review history of their article (what does this mean?). If published, this will include your full peer review and any attached files.

If you choose “no”, your identity will remain anonymous but your review may still be made public.

**Do you want your identity to be public for this peer review?**  For information about this choice, including consent withdrawal, please see our Privacy Policy.

Reviewer #1: No

Reviewer #2: No

Reviewer #3: **Yes:** Junaid Sarfraz

**********

[NOTE: If reviewer comments were submitted as an attachment file, they will be attached to this email and accessible via the submission site. Please log into your account, locate the manuscript record, and check for the action link "View Attachments". If this link does not appear, there are no attachment files.]

To ensure your figures meet our technical requirements, please review our figure guidelines: https://journals.plos.org/plosone/s/figures

You may also use PLOS’s free figure tool, NAAS, to help you prepare publication quality figures: https://journals.plos.org/plosone/s/figures#loc-tools-for-figure-preparation.

NAAS will assess whether your figures meet our technical requirements by comparing each figure against our figure specifications.

# Author comment: Author response to Decision Letter 2

**DOI:** 10.1371/journal.pone.0350264.r005

11 May 2026

Response to Reviewers’ Comments

Manuscript: Forecasting COVID-19 New Cases Using N-BEATS Deep Learning and Mobility Data

Dear Editor and Reviewers,

We sincerely thank the Editor and the Reviewer for the careful evaluation of our manuscript and for the constructive comments. In this revised response, we address the additional comments raised by Reviewer #2 in the latest review round. Reviewer comments are reproduced in italics, followed by our responses describing the revisions and clarifications made in the manuscript.

Reviewer #2

Reviewer #2 – Comment 1

The datasets used by the authors mainly focus on the early stages of the pandemic and lack data on key factors such as vaccination and herd immunity. This may limit the applicability and predictive accuracy of the model in the later stages of the pandemic. It is recommended that the authors provide a detailed discussion in the discussion section on the specific impact of these data limitations on the model's predictive performance and explore how to overcome these limitations in future research.

Response: We thank the reviewer for this important observation. In the revised manuscript, we have added a dedicated discussion paragraph clarifying that the datasets used in this study mainly represent the earlier phases of the COVID-19 pandemic, before vaccination coverage, booster uptake, herd immunity, and variant-specific effects became increasingly important drivers of case dynamics. We now explain that, as a consequence, the relationship learned by the model between mobility patterns and daily new cases is likely to be more reliable for early-wave dynamics than for later pandemic stages. We further note that, in later phases, case trends were increasingly shaped by vaccination, waning immunity, emerging variants, testing practices, and changing public-health interventions, which may reduce predictive accuracy and limit temporal generalizability. Finally, we added future-work directions indicating that vaccination-related, immunity-related, variant-related, testing, and policy-intervention variables should be incorporated in subsequent studies to improve robustness across evolving epidemic phases.

Reviewer #2 – Comment 2

Although the authors reported indicators such as RMSE and MAPE, they did not provide statistical validation such as confidence intervals, statistical tests, or significance levels. It is recommended that the authors conduct dependence-corrected statistical tests to more comprehensively evaluate model performance.

Response: We thank the reviewer for this comment. We agree that the previous version of the manuscript did not provide formal statistical validation such as confidence intervals, significance levels, or dependence-corrected forecast-comparison tests. In the revised manuscript, we have clarified that the reported RMSE and MAPE values are based on experiments repeated five times with different random seeds, and that the reported results correspond to averages across runs. Accordingly, we have revised the manuscript to avoid overstating statistical significance and now describe the results as showing consistent empirical performance improvements rather than formally confirmed statistical significance. In particular, we revised the Abstract and Conclusion to replace significance-based wording with more precise comparative language. We agree that dependence-corrected statistical testing would further strengthen the evaluation, and we identify this as an important direction for future work.

Reviewer #2 – Comment 3

The authors used a trial-and-error approach in selecting model parameters but did not elaborate on this process. It is recommended that the authors provide a more detailed description of hyperparameter optimization, including how to determine the optimal parameter combination and whether cross-validation or other methods were used.

Response: We appreciate the reviewer’s helpful suggestion. We agree that the earlier description of hyperparameter selection was not sufficiently detailed. In the revised manuscript, we clarify that parameter selection was performed using a targeted trial-and-error procedure rather than exhaustive search, and that input chunk length and forecast time-step settings were examined because they had the strongest influence on predictive performance. Candidate settings were assessed using RMSE, MAPE, and training loss, and the final configuration was selected based on overall predictive performance. These additions improve the transparency of the model-selection procedure.

Attachment Submitted filename: Response_to_Reviewers_PLOSONE_COVID_R2_final.pdf

# Editor report: Decision Letter 2

**DOI:** 10.1371/journal.pone.0350264.r006

**Authors:** Jie Zhang

12 May 2026

Forecasting COVID-19 New Cases Using NBEATS Deep Learning and Mobility Data

PONE-D-25-27036R2

Dear Dr. Jallad,

We’re pleased to inform you that your manuscript has been judged scientifically suitable for publication and will be formally accepted for publication once it meets all outstanding technical requirements.

Within one week, you’ll receive an e-mail detailing the required amendments. When these have been addressed, you’ll receive a formal acceptance letter and your manuscript will be scheduled for publication.

An invoice will be generated when your article is formally accepted. Please note, if your institution has a publishing partnership with PLOS and your article meets the relevant criteria, all or part of your publication costs will be covered. Please make sure your user information is up-to-date by logging into Editorial Manager at Editorial Manager® and clicking the ‘Update My Information' link at the top of the page. For questions related to billing, please contact billing support.

If your institution or institutions have a press office, please notify them about your upcoming paper to help maximize its impact. If they’ll be preparing press materials, please inform our press team as soon as possible -- no later than 48 hours after receiving the formal acceptance. Your manuscript will remain under strict press embargo until 2 pm Eastern Time on the date of publication. For more information, please contact onepress@plos.org.

Kind regards,

Jie Zhang

Academic Editor

PLOS One

Additional Editor Comments (optional):

The authors have adequately addressed the reviewers' comments.

Reviewers' comments:

# Editor report: Acceptance letter

**DOI:** 10.1371/journal.pone.0350264.r007

**Authors:** Jie Zhang

PONE-D-25-27036R2

PLOS One

Dear Dr. Jallad,

I'm pleased to inform you that your manuscript has been deemed suitable for publication in PLOS One. Congratulations! Your manuscript is now being handed over to our production team.

At this stage, our production department will prepare your paper for publication. This includes ensuring the following:

* All references, tables, and figures are properly cited

* All relevant supporting information is included in the manuscript submission,

* There are no issues that prevent the paper from being properly typeset

You will receive further instructions from the production team, including instructions on how to review your proof when it is ready. Please keep in mind that we are working through a large volume of accepted articles, so please give us a few days to review your paper and let you know the next and final steps.

Lastly, if your institution or institutions have a press office, please let them know about your upcoming paper now to help maximize its impact. If they'll be preparing press materials, please inform our press team within the next 48 hours. Your manuscript will remain under strict press embargo until 2 pm Eastern Time on the date of publication. For more information, please contact onepress@plos.org.

You will receive an invoice from PLOS for your publication fee after your manuscript has reached the completed accept phase. If you receive an email requesting payment before acceptance or for any other service, this may be a phishing scheme. Learn how to identify phishing emails and protect your accounts at https://explore.plos.org/phishing.

If we can help with anything else, please email us at customercare@plos.org.

Thank you for submitting your work to PLOS ONE and supporting open access.

Kind regards,

PLOS ONE Editorial Office Staff

on behalf of

Dr. Jie Zhang

Academic Editor

PLOS One
